# OneBoot GRU: Validation-Locked Polynomial Calibration for Level-Invariant Encrypted Inference

**DOI:** 10.3390/e28101118

**Published:** 2026-10-13

**Authors:** Xinxia Song, Zhigang Chen, Liqun Chen

**Affiliations:** 1 School of Big Data and Software Engineering, Zhejiang Wanli University, Ningbo 315100, China; 2 College of Artificial Intelligence, Ningbo University of Finance and Economics, Ningbo 315175, China; 3 Department of Computer Science, University of Surrey, Guildford GU2 7XH, UK

**Keywords:** homomorphic encryption, CKKS, gated recurrent unit, polynomial domain calibration, trajectory fidelity, Bootstrapping

## Abstract

Encrypted recurrent inference must preserve both ciphertext depth and the numerical behavior of a feedback model. We develop OneBoot GRU, which couples the validation-locked selection of degree-three Sigmoid and degree-seven Tanh domains with a CKKS GRU cell that refreshes one hidden-state ciphertext after each step. On TwoPatterns, the locked polynomial model completed finite official-test trajectories with 92.431% macro accuracy, versus 98.114% for exact activations, and 0.830% mean domain violations. On SyntheticControl, polynomial and exact macro accuracies were 89.067% and 90.133%. Direct state-error comparisons show that improved domain coverage does not always reduce recurrent error; in a separate exploratory domain extension, screening reduced qualification tail error by 74.1% and 94.6% on ECG200 and ItalyPowerDemand. A parameter profile audited against pinned 128-bit sparse-secret references completed four encrypted recurrent steps, prefix pooling, and the trained head with maximum logit error 2.91 × 10^−6^ relative to the plaintext polynomial circuit. In three matched four-step runs, GRU median circuit latency was 226.343 s versus 336.981 s for an implemented polynomial LSTM. Twelve additional distinct 128-step inputs were completed under an intentionally insecure structural profile, with 12/12 prediction agreement against the plaintext polynomial reference. These experiments provide measured calibration, arithmetic-fidelity, and system-cost operating points within their stated parameter and sampling scopes.

## 1. Introduction

Homomorphic encryption (HE) allows a remote service to evaluate a model without learning its plaintext inputs. The Cheon–Kim–Kim–Song (CKKS) scheme supports approximate packed arithmetic and provides a basis for evaluating neural networks through polynomial operations [1]. For sequential data, the computation must also preserve information across time. A recurrent model passes its computed hidden state to the next step, so an approximation error can change the inputs received by every subsequent activation. A ciphertext can remain computationally usable while the model’s numerical trajectory departs from the intended computation.

This paper studies how to select Sigmoid and Tanh approximation domains for a fixed-degree gated recurrent unit (GRU) and assesses the resulting model before its official test data are used. The practical risk is accepting a polynomial model on the basis of an incomplete indicator of reliability. High classification accuracy can coexist with repeated evaluation outside the fitted activation domains. Low domain violation rates can coexist with substantial state errors or lost predictive accuracy. Successful ciphertext refresh only restores the depth available for further computation. A useful assessment must distinguish these properties and identify which requirements a candidate actually satisfies.

The difficulty arises from the interaction between scalar approximation and recurrent feedback. A narrow fitting interval can improve the approximation of an activation on that interval while excluding values reached by the model. A broader interval can cover more preactivations while increasing approximation error at the same degree. In a GRU, the update and reset gates affect the hidden state and the candidate input, so changing either polynomial also changes later values presented to both functions [2]. Separate scalar fits cannot determine the behavior of the complete recurrent model. The relevant evaluation unit is a pair of domains applied to complete trajectories with fixed weights.

Candidate selection also creates an evaluation risk. Polynomial coefficients are part of the executable model even when the neural weights remain unchanged. Adjusting their domains in response to official test failures therefore uses the test set for model development. OneBoot GRU addresses this risk by separating model fitting, candidate selection, qualification, and test access. Candidate pairs are assessed on ordinary trajectories and deterministic longer repetitions. A domain-constrained rule combines observed finiteness with an explicit domain threshold and an accuracy requirement. The chosen weights, coefficients, and assessment rules are locked before a disjoint qualification sample is evaluated. Qualification checks rank coverage, ordinary and stress finiteness, and task utility separately, without substituting one condition for another.

The reason for this separation is operational. Full trajectory evaluation exposes failures that isolated activation fits cannot reveal. Locking the candidate prevents qualification or test outcomes from silently changing the model, and separate checks make the cause of rejection explicit. These properties improve the interpretability of an assessment. They do not imply that imposing a domain constraint improves the model’s dynamics. Direct hidden state discrepancies and complete GRU Jacobian products are therefore used to examine the relationship between domain coverage and recurrent error. A population interpretation of the qualification rank additionally requires an appropriate sampling model, including exchangeability with future scores [3,4].

The encrypted implementation provides the computational setting for this assessment. Bootstrapping restores the level budget required by another recurrent transition [5]. The privacy-preserving long short-term memory (PPLSTM) protocol combines repeated CKKS refresh with normalization-based range control for long short-term memory (LSTM) inference [6]. A GRU has one persistent recurrent state and permits a refresh boundary after its complete update. The cell studied here uses degree 3 gate polynomials and a degree 7 candidate polynomial, consuming eight levels before refresh. Restoring the same budget after each step supports a level invariant schedule.

The experiments establish concrete operating points for the fixed-degree design. On TwoPatterns, the selected polynomial model has finite official-test trajectories, a mean domain violation rate of 0.830% and macro accuracy of 92.431%, against 98.114% for exact activations. On SyntheticControl, the prospectively selected polynomial model attains 89.067% macro accuracy, compared with 90.133% for its exact reference, with finite ordinary and doubled-length test trajectories. The protocol also records rejected candidates explicitly, making its acceptance criteria inspectable across datasets.

The same-model comparisons establish complementary roles for coverage, direct trajectory error and sensitivity diagnostics. Four additional archive tasks broaden the original two-task prospective comparison. In a separately labeled exploratory domain-grid extension, domain-aware selection reduces qualification tail state error by 74.1% on ECG200 and 94.6% on ItalyPowerDemand while improving macro accuracy by 1.25 and 2.50 percentage points. Across 490 candidate–seed analyses on the original two tasks, direct selection error strongly predicts qualification error. These results identify settings in which coverage screening is useful and retain direct fidelity measurement as a distinct requirement.

Encrypted validation covers two complementary scopes. The four-step target-profile run completes actual recurrent refresh, prefix pooling, and the trained head; three matched repetitions measure GRU and polynomial-LSTM system cost with the same cryptographic parameters and hardware. Twelve additional distinct 128-step sequences test long-horizon arithmetic and descriptive error distributions under the smaller, intentionally insecure structural profile. The former supports short-run execution with parameters audited against pinned 128-bit references; the latter supports long-run numerical verification but makes no security claim. Section 7.10 and Section 7.16 report these outcomes separately.

The contributions of this paper are threefold:

(1) We formulate a validation-locked protocol for the coupled selection of Sigmoid and Tanh approximation domains in a fixed-degree GRU. The protocol separates model fitting, trajectory-based candidate selection, qualification, and test access, and evaluates domain coverage, ordinary and extended horizon finiteness, and task utility as distinct requirements.
(2) We provide the complete GRU Jacobian analysis and an eight-level encrypted recurrence schedule with one hidden state refresh per step. These elements connect recurrent sensitivity analysis with an executable-level invariant CKKS circuit while making clear that the schedule supplies no global hidden state bound.
(3) We evaluate calibration, recurrent fidelity, and encrypted execution with complementary controls: polynomial-degree and threshold sensitivity, 490 candidate–seed Jacobian analyses, four additional datasets, gate-level interventions, twelve new complete encrypted sequences, and a matched target-profile GRU–LSTM comparison. The observed improvements are reported together with task accuracy and direct state error. Frozen protocols, checkpoint identities, raw traces, and separate exploratory labels make the scope of each comparison inspectable.

## 2. Related Work

### 2.1. Homomorphic Recurrent Inference

CKKS supports approximate packed arithmetic, while Bootstrapping restores a depleted ciphertext level budget [1,5]. CryptoRNN and noninteractive recurrent prediction established early approaches to encrypted recurrence using approximated activations [7,8]. Recurrent evaluation differs from a fixed feedforward graph because numerical error changes inputs to later copies of the same cell. A schedule that restores depth therefore still requires an assessment of the resulting approximate dynamics.

GRU maintains update and reset gates with one persistent recurrent state [2]. CryptoGRU combines homomorphic linear operations with garbled circuit nonlinear evaluation, giving a different interaction model from pure CKKS inference [9]. MatHEGRU uses a specialized matrix homomorphic representation for sequential computation [10]. Wang and Ikeda study high-throughput encrypted GRU and a later bootstrapping-free text classification design [11,12]. Avoiding refresh is attractive when the selected modulus chain covers a fixed short horizon. The present schedule instead pays for a refresh after each cell, making the recurrent depth budget independent of the processed prefix.

### 2.2. LSTM Systems and the Role of PPLSTM

LSTM carries both a cell state and a hidden state [13]. LSTM building blocks based on Fast Fully Homomorphic Encryption over the Torus (TFHE), PrivLSTM, and PPLSTM investigate different encrypted realizations of this structure [6,14,15]. PPLSTM is the closest system reference here because it combines continuing CKKS recurrence, explicit refresh scheduling, packed linear algebra, and normalization-based range control.

PPLSTM refreshes the two values propagated between its LSTM rounds and uses root mean square layer normalization (RMSNorm) to control values entering nonlinear approximation. The present GRU has one persistent recurrent state and omits that normalization path. Its complete transition fits an eight-level residual circuit. Section 7.10 tests the architectural cost with an implemented polynomial LSTM using shared CKKS kernels, parameters, packing and hardware. This matched baseline quantifies one-versus-two-state refresh scheduling; it is a standard LSTM comparison, not a reproduction of PPLSTM’s text model and RMSNorm pipeline.

The comparison separates computation from model qualification. Both trained recurrent architectures are evaluated with exact and polynomial activations, and their task utility is reported alongside encryption cost. This provides an executable system baseline while preserving PPLSTM as the reference for normalization-based range control. Domain screening, direct fidelity and task accuracy remain separately assessed properties.

### 2.3. Polynomial Adaptation and Domain Selection

Low-degree polynomials reduce sequential multiplication requirements but can incur substantial approximation error over broad intervals. Their evaluation can use depth-efficient multiplication schedules [16]. In recurrent models, changing the approximation interval also changes later activation inputs, which makes scalar approximation error insufficient for assessing the complete computation.

Polynomial adaptation is established in private inference. Accuracy Preserving Low-degree Polynomial Activation (AESPA) uses a Hermite-based activation and basis-wise normalization to improve the accuracy of low-degree models in a hybrid private inference setting [17]. AutoFHE, an automated fully homomorphic encryption (FHE) adaptation framework, jointly optimizes mixed-degree polynomial activations and Bootstrapping placement for encrypted convolutional networks [18]. These works show that activation adaptation and accuracy cost co-optimization are prior concepts. The present study fixes the degree and recurrent schedule and evaluates coupled Sigmoid and Tanh domains through complete trajectories. It contributes a recorded decision procedure and its empirical trade-offs, without claiming general polynomial adaptation novelty or superiority over those different architectures.

The relevant costs differ across these approaches: scalar coefficient fitting, model adaptation, domain search and encrypted execution should not be conflated. AESPA concerns low-degree activation design and normalization [17], whereas AutoFHE jointly adapts mixed degrees and refresh placement for convolutional neural networks (CNNs) [18]. Our offline search holds degrees and neural weights fixed. Section 4.8 accounts separately for fitting, trajectory screening and Jacobian diagnostics, and Section 7 reports measured encrypted components within their parameter scope.

The secondary fine-tuning experiment similarly builds on existing training principles. Classification and softened teacher targets provide a matched ordinary adaptation control [19]. A trajectory maximum penalty supplies the additional treatment. The comparison concerns its incremental behavior for the fixed polynomial GRU.

### 2.4. Statistical Qualification and Recurrent Diagnostics

Split conformal methods obtain marginal coverage under exchangeability, while tolerance procedures concern population content at a specified confidence level [3,4,20]. These statements require different interpretations. A disjoint qualification sample prevents direct reuse for candidate selection, but a fixed class allocation does not automatically supply exchangeability with a future population sample. The rank check in the present assessment is therefore reported as an empirical screen unless its population assumptions can be justified. The distinction between theoretical assumptions and observed pass decisions is part of the methodological scope.

Products of recurrent Jacobians describe local propagation of gradients and state perturbations [21,22]. For a polynomial GRU, the derivative includes update and reset gates as well as the candidate’s indirect dependence on the reset gate. We give the complete expression and verify it numerically.

### 2.5. Position of This Study

Table 1 positions this work within encrypted recurrent inference. It asks what accuracy and finiteness checks miss when polynomial domains are chosen, how an explicit domain constraint changes that decision, and whether the selected model can be connected to a working encrypted arithmetic implementation. The same grid replay identifies the domain constraint’s effect on the observed selection. Locked test access preserves the distinction between development decisions and their later evaluation. The complete Jacobian and repeated refresh circuit support interpretation and implementation. The direct error comparisons further test whether domain coverage predicts actual recurrent fidelity.

## 3. Background and Problem Formulation

### 3.1. CKKS Encrypted Arithmetic

The Cheon–Kim–Kim–Song scheme, commonly known as CKKS, is an approximate homomorphic encryption scheme designed for computations over real or complex numbers. CKKS encodes a vector of numerical values into a polynomial in the quotient ring $R_{Q}=Z_{Q}[X]/(X^{N}+1)$, where N is a power of two and Q is a ciphertext modulus. A client encrypts the encoded polynomial under a public key and sends the resulting ciphertext to a server. The server can evaluate additions and multiplications directly on ciphertexts, while the client retains the secret key required for decryption. The decrypted output approximates the corresponding plaintext computation because CKKS represents numerical values with a finite scale and introduces small approximation errors during encoding and homomorphic evaluation.

CKKS supports packed computation through its single instruction, multiple data (SIMD) capability. A single ciphertext can contain many numerical values, and the same arithmetic operation is applied to all packed slots in parallel. Slot rotations move packed values across positions and enable sums, inner products, and matrix-vector products. In an encrypted recurrent network, the input vectors and hidden states can therefore be arranged so that several samples or hidden dimensions share one ciphertext. Plaintext ciphertext matrix multiplication can then be implemented through diagonal encoding, rotations, and plaintext multiplications. This packed representation amortizes the cost of linear transformations across many values and has a direct influence on throughput, rotation key storage, and memory consumption.

Each ciphertext is associated with a scale and a position in a modulus chain. A ciphertext multiplication increases the scale and consumes part of the available modulus. Rescaling reduces the scale and removes one modulus prime, which lowers the remaining level of the ciphertext. Relinearization controls ciphertext size after multiplication, while modulus alignment ensures that operands have compatible levels before an addition or multiplication. These procedures allow CKKS to evaluate a bounded depth arithmetic circuit. A recurrent network creates an additional difficulty because the output state of one time step becomes an input to the next time step. Repeating the same cell therefore consumes additional levels until the available modulus chain is exhausted.

Bootstrapping restores the computational capacity of a ciphertext by homomorphically evaluating an approximate decryption and modulus reduction procedure [5]. After Bootstrapping, the ciphertext returns to a high level and can participate in further multiplications. The operation also introduces a small numerical error that becomes part of the encrypted inference error. Bootstrapping requires large evaluation keys and substantially more computation than addition, multiplication, rescaling, or rotation. Its frequency consequently determines much of the latency and memory cost of an encrypted recurrent model. A design that refreshes the recurrent state after every time step can process a continuing stream because each new iteration starts with a refreshed state. The total running time remains linear in the number of time steps.

For the implementation studied here, write (pk, sk, evk) ← KeyGen(κ, params), where κ denotes a target security category and params fixes the ring, the full ciphertext and auxiliary modulus chains, default scale, secret and error distributions, and the Bootstrapping configuration. Parameter selection precedes key generation. The client samples and retains sk, derives pk, and generates the relinearization, rotation, ring-switching, and Bootstrapping evaluation keys needed by the declared circuit; the server receives pk and the required evk but not sk. The target κ = 128 is supported here by a version-pinned sparse-secret reference audit, not by a fresh attack-by-attack estimate for the exact parameter tuple. Let E denote vector encoding at scale Δ and D denote decoding. For each transformed input,

(1) $$c_{t}^{x}={Enc}_{pk}(E_{\Delta }({\tilde{x}}_{t})),\;c_{0}^{h}={Enc}_{pk}(E_{\Delta }(0)).$$

The server’s step is the following arithmetic evaluation followed by refresh:

(2) $${\tilde{c}}_{t}^{h}={Eval}_{evk}(F_{\theta }^{pol},c_{t}^{x},c_{t-1}^{h}),$$

(3) $$c_{t}^{h}={Boot}_{evk}({\tilde{c}}_{t}^{h}).$$

Decoding the decrypted state gives the polynomial recurrence plus the CKKS perturbation formalized in Section 3.4 and Section 5.1. The model-to-arithmetic correspondence is explicit: public weights and packed diagonal encodings implement the six affine paths through plaintext–ciphertext products, rotations, and sums; ciphertext products evaluate polynomial powers, the reset product, and the final hidden-state interpolation; rescaling and level alignment keep those operands compatible. The hidden-state ciphertext is bootstrapped after the complete update, then reused by the next time step and the encrypted temporal accumulator. The trained linear head acts on encrypted pooled states. The client decrypts the returned logits and applies argmax; neither an encrypted comparison nor an encrypted softmax is part of this inference circuit. Selecting different fitting domains changes the public polynomial coefficients and visited states while preserving this fixed-degree arithmetic graph.

CKKS can evaluate polynomial functions using additions and multiplications. Neural networks contain functions such as Sigmoid and Tanh that fall outside this arithmetic interface. Encrypted inference systems replace them with polynomial approximations over a prescribed interval. A narrow interval usually allows a low-degree polynomial to achieve a small approximation error. A wider interval improves coverage of activation inputs and often increases the approximation error when the degree remains fixed. Increasing the polynomial degree can improve approximation quality, although it also raises multiplicative depth and may require additional Bootstrapping. The choice of approximation interval therefore affects numerical accuracy, circuit depth, and recurrent stability at the same time.

### 3.2. Gated Recurrent Unit Inference

A GRU updates a hidden vector using update and reset gates [2]. We use a custom reset-after-linear cell, defined below, rather than assuming that all library GRU variants are identical. Let x_t_ ∈ R^d^, h_t−1_ ∈ R^m^, Wg ∈ R^m×d^, Ug ∈ R^m×m^ and bg ∈ R^m^ for g ∈ {z, r, n}; the initial state is h_0_ = 0. The exact-activation transition is

(4) $$a_{t}^{z}=W_{z}x_{t}+U_{z}h_{t-1}+b_{z},\;z_{t}=\sigma (a_{t}^{z})$$

(5) $$a_{t}^{r}=W_{r}x_{t}+U_{r}h_{t-1}+b_{r},\;r_{t}=\sigma (a_{t}^{r})$$

(6) $$a_{t}^{n}=W_{n}x_{t}+r_{t}⊙(U_{n}h_{t-1})+b_{n},\;n_{t}=tanh(a_{t}^{n})$$

All activations and Hadamard products are elementwise. The implementation assigns biases to the input linear layers and sets both recurrent linear layers to bias = False. In particular, there is no recurrent candidate bias inside the reset product. Applying the reset gate after U_n_h_t−1_ is part of this model definition; moving it inside the matrix product would define a different cell. The exact teacher, polynomial evaluator, analytical Jacobian and exported encrypted circuit use this same convention.

The hidden-state update and its algebraically equivalent encrypted evaluation form are

(7) $$h_{t}=(1-z_{t})⊙n_{t}+z_{t}⊙h_{t-1}=n_{t}+z_{t}⊙(h_{t-1}-n_{t}).$$

This equivalent form provides a convenient circuit because it expresses the final update with one elementwise ciphertext multiplication after the candidate state is available. The affine transformations for the update gate, reset gate, and candidate state contain an input path and a recurrent path. The encrypted cell consequently evaluates six packed plaintext ciphertext matrix multiplications. The remaining operations consist of polynomial activation evaluation, elementwise multiplication, addition, subtraction, rescaling, and a final state refresh.

The GRU carries one persistent vector, $h_{t}$, from one time step to the next. An LSTM carries both a cell state $c_{t}$ and a hidden state $h_{t}$. This distinction has an important cryptographic consequence. In an encrypted LSTM designed for continuing inference, both persistent states must retain enough computational capacity for the following iteration. PPLSTM refreshes the cell state and the hidden state within each recurrent round and uses placement optimization to reduce the cost of this two-state schedule. A GRU offers a different circuit structure because the complete recurrent memory is represented by a single ciphertext state. Once the gate computations and hidden update are complete, one Bootstrapping operation can refresh $h_{t}$ for the next time step.

The single persistent state permits one logical refresh boundary after the cell. Numerical reliability remains a separate requirement: polynomial replacements alter gate outputs and the hidden state supplied to later steps. Even inside a fitting interval, an unconstrained least-squares Sigmoid polynomial need not take values in [0, 1]. Consequently, interval containment alone does not preserve the convex-combination interpretation of the exact update. Section 5 evaluates this issue through direct state error and the complete recurrent derivative.

### 3.3. System and Threat Model

The system contains a client and a model server. The client owns a private input sequence $X=(x_{1},\ldots ,x_{T})$ and generates the CKKS secret key, public key, and evaluation keys. The evaluation keys include the relinearization keys, rotation keys, and Bootstrapping keys required by the encrypted circuit. The client preprocesses the sequence according to the public input specification, encodes the resulting features, encrypts them under the public key, and transmits the ciphertexts to the server. Preprocessing may include public normalization rules, clipping to a fixed interval, and causal difference features. These transformations are performed before encryption and remain deterministic parts of the inference protocol.

The server holds the trained GRU parameters in plaintext. It receives the encrypted sequence and evaluates the recurrent cell using CKKS operations. At every time step, the server computes the encrypted gates, candidate state, and hidden state. It then performs one Bootstrapping operation on the updated hidden state before processing the next input. After the final time step, the server applies the encrypted pooling and classification head and returns an encrypted prediction to the client. The client decrypts the output and obtains the approximate model result.

The server is honest but curious: it executes the prescribed circuit while trying to infer the client’s input from the ciphertexts, public parameters, and evaluation keys. Input confidentiality relies on the RLWE-based security of the selected CKKS parameters [23] under this model. The client alone holds the secret key; the server receives no plaintext input or intermediate state during deployment. The model architecture and weights, cryptographic parameters, packing layout, and processed sequence length are public. A deployment requiring length privacy must pad to a public upper bound. The sparse-secret parameter audit in Section 6.8 and Section 7.7 evaluates reference-profile conformance; successful encryption or Bootstrapping alone is not evidence of a particular security level.

The protection claimed here is client-input confidentiality during outsourced inference. Model confidentiality, malicious-client input validation, active server deviation, denial of service, and inference from repeated outputs are outside the evaluated guarantee. The client is expected to apply the public clipping and feature transformation before encryption. Intermediate states are decrypted in controlled experiments only to measure arithmetic error; those decrypted values are never sent to the server or fed back into encrypted recurrence. The deployed circuit requires no intermediate decryption. These assumptions identify precisely what the experiments measure and what additional controls an application would need.

### 3.4. Encrypted Recurrent Computation

Let $f_{\theta }$ denote a trained GRU model with parameters θ. Plaintext inference applies an exact recurrent map $F_{\theta }$ at each time step according to

$$h_{t}=F_{\theta }\;\left(x_{t},h_{t-1}\right),\;h_{0}=0.$$

Encrypted inference replaces Sigmoid and Tanh with polynomial approximations $p_{\sigma }$ and $p_{tanh}$. The resulting polynomial recurrent map is denoted by ${\tilde{F}}_{\theta }$. Let Enc, Dec, and Bts denote CKKS encryption, decryption, and Bootstrapping. Given encrypted inputs $ct(x_{t})$, the server evaluates

$$ct(u_{t})={Eval}_{{\tilde{F}}_{\theta }}\;(ct(x_{t}),ct({\hat{h}}_{t-1})),$$

$$followed\;by\;ct({\hat{h}}_{t})=Bts\;\left(ct(u_{t})\right).$$

The decrypted state can be expressed as

$${\hat{h}}_{t}={\tilde{F}}_{\theta }\;(x_{t},{\hat{h}}_{t-1})+\varepsilon_{t}^{ckks},$$

where $\varepsilon_{t}^{ckks}$ represents the accumulated CKKS error from encoding, rescaling, key switching, polynomial evaluation, and Bootstrapping during time step t. The difference between $F_{\theta }$ and ${\tilde{F}}_{\theta }$ contributes an additional approximation error. Both components enter the next recurrent state and can influence subsequent computation.

Cryptographic continuation is defined through a level invariant. After every Bootstrapping operation, $ct({\hat{h}}_{t})$ returns to a designated level and scale that are sufficient for one complete evaluation of ${\tilde{F}}_{\theta }$. This invariant is independent of t. It removes exhaustion of the initial modulus chain as a horizon limit, while total latency grows proportionally with T. Numerical continuation requires a separate domain and stability argument and is evaluated only over the stated data and stress distributions.

At the level of one complete step, the server evaluates three packed affine gate inputs from the fresh encrypted feature vector and the preceding refreshed hidden-state ciphertext. It applies the locked cubic gate polynomial twice, forms the reset-weighted candidate input, applies the degree-seven candidate polynomial, and computes the hidden interpolation. Only the final hidden-state ciphertext crosses the recurrent boundary; one Bootstrapping call restores its depth and approximate default scale before the next input. Pooling receives that refreshed state through ciphertext additions, and the trained linear head is evaluated after the requested horizon. Equations (4)–(7) define the plaintext map, while this encrypted schedule defines how the same fixed polynomial map is executed under CKKS. The repeatable level cycle supports arithmetic continuation; finite trajectories, state error, and task utility are assessed separately over the stated data and horizons.

### 3.5. Polynomial Domain and Stability Conditions

Let $D_{\sigma }=[-B_{\sigma },B_{\sigma }]$ and $D_{tanh}=[-B_{tanh},B_{tanh}]$ denote the fitting domains of the Sigmoid and Tanh polynomials. The update and reset preactivations should remain within $D_{\sigma }$, and the candidate preactivation should remain within $D_{tanh}$ for the approximation errors to match their calibrated values. Exact containment over every possible input can be overly restrictive for learned recurrent models. We therefore use a data-dependent calibration condition that evaluates complete polynomial trajectories on a held-out calibration set and on an extended length stress set.

For a calibration collection C, define $N_{nf}(B_{\sigma },B_{tanh},C)$ as the number of trajectories that contain a nonfinite hidden state or output under a jointly fitted polynomial pair. An eligible pair satisfies $N_{nf}(B_{\sigma },B_{tanh},C)=0\;and\;N_{nf}(B_{\sigma },B_{tanh},C^{stress})=0,$ where $C^{stress}$ contains deterministic repetitions with a longer recurrent horizon. Domain-constrained eligibility also requires the joint trajectory violation rate to remain below a frozen per-checkpoint threshold. Candidate selection uses declared accuracy and tie-breaking rules. A disjoint qualification subset then evaluates the selected pair through separate rank coverage, ordinary finiteness, stress finiteness, and utility checks. A statistical interpretation of the rank requires exchangeability with future scores and is not supplied by disjointness alone. We therefore distinguish an empirical assessment from a population guarantee. The selected pair, assessment outcome, checkpoint identities, and protocol are locked before official test evaluation.

Long-horizon behavior is further characterized through the hidden state Jacobian. For the polynomial recurrent map at time t, define

$$J_{t}=\frac{\partial {\tilde{F}}_{\theta }\;\left(x_{t},h_{t-1}\right)}{\partial h_{t-1}}.$$

The propagation of a small state perturbation over k time steps is governed locally by the product

$$J_{t:t-k+1}=J_{t}J_{t-1}\cdots J_{t-k+1}.$$

The spectral norm $\|J_{t:t-k+1}{\|}_{2}$ measures local amplification along an observed trajectory. Individual values of $\|J_{t}{\|}_{2}$ may exceed one because the GRU is an input-driven and time-varying system. A multi-step product below one indicates contraction over the corresponding observed segment. These measurements provide empirical evidence about error propagation along evaluated trajectories. A global stability certificate would additionally require an invariant domain and a uniform bound covering every admissible input and hidden state.

### 3.6. Problem Statement

The screening objective is to determine, before test access, whether a fixed-degree polynomial GRU satisfies a stated set of empirical qualification rules and supports a repeatable CKKS level schedule. Given fixed weights, frozen candidate domains and disjoint training-derived partitions, the procedure selects a pair or reports that no candidate meets the declared requirements. A qualified model must separately satisfy its numerical and task conditions. A research gate pass is neither cryptographic security evidence nor deployment approval. Table 2 defines the distinct properties assessed in this paper.

Let $A_{exact}$ denote the task accuracy of the exact activation model and let $A_{poly}$ denote the accuracy of the polynomial model under the locked evaluation protocol. Task fidelity is expressed through

$$A_{poly}\geq A_{exact}-\epsilon_{acc},$$

where $\epsilon_{acc}$ is an acceptable loss specified before the relevant utility evaluation. A comparison with the exact model does not establish absolute task usefulness when that model is weak. Direct hidden state error is evaluated separately because prediction agreement can conceal trajectory differences. Accuracy cannot override a failed finite trajectory requirement, and every nonfinite trajectory is counted as an incorrect prediction. The finite trajectory requirement is written as

$${\hat{h}}_{t}\in R^{m}\;for\;all\;t\in \{1,\ldots ,T\}$$

for every evaluated sequence, with an explicit numerical finite check on states and outputs. Qualification applies the corresponding finite checks to ordinary and matched stress trajectories independently of the rank threshold. These checks do not create test authorization. A separately declared research policy can evaluate all locked variants, including rejected ones, without approving them for deployment. The rules used by each experiment are identified in Section 6.13.

The cryptographic requirement is expressed through a recurrent level invariant. If $L_{ref}$ and $\Delta_{ref}$ denote the level and scale after refresh, then each iteration must satisfy

$$Level\;(ct({\hat{h}}_{t}))=L_{ref}$$

$$and\;Scale\;(ct({\hat{h}}_{t}))\approx \Delta_{ref}.$$

The methodological objective is to make unmet requirements and test-dependent model changes explicit before a polynomial model is presented as suitable for deployment. The system objective is to realize the qualified pair with one persistent state refresh per step and a repeatable level schedule. A dataset may fail the qualification protocol even when the encrypted circuit itself is executable. The following section develops the joint calibration protocol and its encrypted realization.

### 3.7. Secondary Question: Fine-Tuning and Requalification

The primary problem in Section 3.6 concerns a fixed model and remains unchanged by the following extension. A secondary question asks whether fine-tuning can reduce domain excursions without changing the selected polynomial coefficients or arithmetic graph. This adaptation is a separate experimental study and is not a fallback that silently reverses a frozen rejection. Let $\theta_{0}$ denote the exact activation teacher parameters and $\theta_{\lambda }$ the parameters after polynomial fine-tuning with domain penalty weight λ. The ordinary polynomial fine-tuning control uses λ=0. Both arms begin from the same teacher and use the same training examples, batch order, and number of updates. This paired design isolates the additional trajectory penalty.

For a finite trajectory, let $S_{i}(\theta )$ denote its largest normalized preactivation across all times, coordinates, and gate paths. Define V(θ) as the probability of $S_{i}>1$, counting any nonfinite trajectory as a violation, and F(θ) as the probability of nonfinite recurrence. A practical repair objective seeks to lower V with a specified task accuracy tolerance and no increase in F. These population quantities are unknown. The experiments report empirical comparisons and separate qualification statistics. No population improvement theorem or formal accuracy noninferiority result is claimed.

A fine-tuned checkpoint remains subject to independent assessment of domain coverage, finite ordinary and stress trajectories, and task utility. The disjoint qualification data must remain unused during the development or selection of that checkpoint and its evaluation rule. Changing weights invalidates any automatic transfer of an earlier checkpoint’s qualification. An exploratory improvement on reused qualification data supplies mechanism evidence and cannot create a new independent guarantee.

The target population of successful repairs is an empirical research question. A finite initial polynomial forward pass permits loss and gradient computation and does not ensure that all subsequent updates remain finite. The current penalty cannot recover an undefined loss by itself. The study therefore retains unsuccessful training runs and does not restrict reported transfer outcomes to checkpoints that were observed to succeed.

## 4. Proposed Method

This section presents the proposed OneBoot GRU framework for encrypted sequence inference. The method combines an HE-compatible temporal representation, low-degree polynomial gates, a validation-locked domain calibration procedure, packed linear transformations, and a recurrent circuit that refreshes one hidden state after each time step. The construction is designed for standard CKKS arithmetic and requires no interaction with the client during recurrent evaluation. Section 4.1, Section 4.2, Section 4.3, Section 4.4, Section 4.5, Section 4.6, Section 4.7 and Section 4.8 define the core calibration and execution procedure. Section 4.9 presents a separately evaluated fine-tuning extension with its own model identities and access protocol.

### 4.1. Method Overview

Let $X=(x_{1},\ldots ,x_{T})$ denote a sequence with d input channels and let θ denote the trained GRU parameters. The client first applies the public feature transformation described in Section 4.2. It then packs and encrypts the transformed sequence under CKKS. The server evaluates the polynomial GRU cell for each encrypted input, refreshes the resulting hidden state once, and continues with the next time step. A temporal aggregation module collects the refreshed hidden states through encrypted additions. The server finally evaluates a plaintext ciphertext linear head and returns encrypted logits to the client.

The recurrent cell follows the standard GRU dependency structure introduced in Section 3. The Sigmoid and Tanh functions are replaced by degree 3 and degree 7 polynomials. These degrees allow the complete hidden update to fit within an eight-level residual circuit. Their intervals are selected jointly on a held-out partition and assessed on a separate qualification partition before official test access. Coverage, finite trajectories, and utility are checked separately under the conditions in Section 4.4. The selection rule evaluates complete recurrent trajectories and rejects every pair that produces a nonfinite state or exceeds the frozen domain violation threshold. This trajectory-based procedure accounts for the feedback between approximation error and later gate inputs.

The method separates model learning from deployment calibration. Exact Sigmoid and Tanh functions are used during the main training stage. The trained weights remain fixed while candidate polynomial pairs are evaluated on the selection partition. This separation avoids training a different model for every candidate and makes both selected intervals explicit deployment parameters. A disjoint qualification partition evaluates the locked pair before test access.

### 4.2. HE Compatible Temporal Representation

Time series classification often depends on local changes and the position of a pattern within the observation window. A single running mean of the hidden states can remove this positional information. We therefore construct a temporal representation that exposes changes at several causal scales and preserves coarse temporal location. The design uses client-side clipping, subtraction, encrypted addition, and multiplication by public constants. These operations add no ciphertext nonlinear depth inside the recurrent cell.

For an input channel q, the client clips every value to a public bound C according to

$${\bar{x}}_{t,q}={clip}_{C}(x_{t,q})=min\;\left(C,max\;\left(-C,x_{t,q}\right)\right).$$

The experiments use C=4. Clipping is performed before encryption because comparison-based clipping would require a substantially deeper encrypted circuit. The rule is fixed before training and evaluation. Applying the same rule to the training, calibration, and test partitions keeps the model input distribution consistent with the encrypted deployment interface.

Let L denote a public collection of causal lags. For each ℓ∈L, the client forms a clipped difference channel

$$d_{t,q}^{\left(\ell \right)}=\left\{\begin{array}{cc} 0, & t\leq \ell ,\\ {clip}_{C}\;\left({\bar{x}}_{t,q}-{\bar{x}}_{t-\ell ,q}\right), & t>\ell . \end{array}\right.$$

The deployed configuration uses L={1,2,4,8,16}. The transformed input concatenates the clipped raw value with all difference channels as

$${\tilde{x}}_{t}=\left[{\bar{x}}_{t}\;\|\;d_{t}^{\left(1\right)}\;\|\;d_{t}^{\left(2\right)}\;\|\;d_{t}^{\left(4\right)}\;\|\;d_{t}^{\left(8\right)}\;\|\;d_{t}^{\left(16\right)}\right].$$

The symbol ‖ denotes concatenation. These causal differences provide short- and medium-range change information without introducing future observations. An implementation that receives encrypted raw values can form the differences through ciphertext subtraction. The additional clipping of each difference channel is currently assigned to the client, which ensures that all encrypted input features lie within the declared range.

The recurrent outputs are summarized through temporal pyramid pooling, which adapts hierarchical pooling to a one-dimensional sequence [24]. Let K denote the number of temporal bins and define

$$I_{k}=\left\{t\;|\;\left\lfloor \frac{K(t-1)}{T}\right\rfloor =k-1\right\},$$

where k∈{1,…,K}. The pooled state for bin k is

$$s_{k}=\frac{1}{\left|I_{k}\right|}\sum_{t\in I_{k}}{\hat{h}}_{t}.$$

The final representation is $s=[s_{1}\|\cdots \|s_{K}]$. The experiments use K=8. Each accumulator requires ciphertext additions, and the final average uses multiplication by the public reciprocal $1/|I_{k}|$. The pooling module therefore preserves coarse temporal position with no additional ciphertext multiplication in the recurrent path. For a finite sequence, the bin boundaries are derived from the public length T. A continuing stream can expose a prediction at a requested horizon through the last hidden state or a running mean while retaining the same OneBoot recurrent cell.

### 4.3. Polynomial GRU Cell

The encrypted cell replaces each Sigmoid function with a degree 3 polynomial fitted on a symmetric interval $\left[-B_{\sigma },B_{\sigma }\right]$, defined as $p_{\sigma ,B_{\sigma }}(u)=0.5+a_{1}(B_{\sigma })u+a_{3}(B_{\sigma })u^{3}$.

The coefficients are obtained by unweighted least squares over a dense uniform grid. The fixed constant preserves the identity $p_{\sigma ,B_{\sigma }}(-u)=1-p_{\sigma ,B_{\sigma }}(u)$. Its constant term is encoded as plaintext, and its powers are evaluated with two sequential ciphertext multiplication levels. The same candidate polynomial is applied to the update and reset preactivations.

The Tanh function is replaced by an odd degree 7 polynomial fitted on $\left[-B_{tanh},B_{tanh}\right]$. Its approximation has the form

$$p_{tanh,B_{tanh}}(u)=a_{1}(B_{tanh})u+a_{3}(B_{tanh})u^{3}+a_{5}(B_{tanh})u^{5}+a_{7}(B_{tanh})u^{7}.$$

The coefficients are obtained through least squares fitting on a dense uniform grid over the target interval. If $G_{B_{tanh}}$ denotes this grid, the coefficient vector is defined by

$$a(B_{tanh})=\underset{a\in R^{4}}{argmin}\sum_{u\in G_{B_{tanh}}}{\left(tanh(u)-a_{1}u-a_{3}u^{3}-a_{5}u^{5}-a_{7}u^{7}\right)}^{2}.$$

Odd symmetry removes the even powers and preserves $p_{tanh,B_{tanh}}(0)=0$. The polynomial degree remains fixed across candidate intervals, so interval selection changes the coefficients and approximation error while preserving multiplicative depth. The powers can be evaluated with a depth-efficient multiplication schedule following established low-multiplication polynomial evaluation methods [18]. The resulting degree 7 circuit consumes three levels after its input has been prepared.

Using the notation from Section 3, the polynomial gates are computed as

$${\tilde{z}}_{t}=p_{\sigma ,B_{\sigma }}\;\left(W_{z}{\tilde{x}}_{t}+U_{z}{\hat{h}}_{t-1}+b_{z}\right),$$

$${\tilde{r}}_{t}=p_{\sigma ,B_{\sigma }}\;\left(W_{r}{\tilde{x}}_{t}+U_{r}{\hat{h}}_{t-1}+b_{r}\right),$$

$$and\;{\tilde{n}}_{t}=p_{tanh,B_{tanh}}\;\left(W_{n}{\tilde{x}}_{t}+{\tilde{r}}_{t}⊙\left(U_{n}{\hat{h}}_{t-1}\right)+b_{n}\right).$$

The hidden state before refresh is ${\tilde{h}}_{t}={\tilde{n}}_{t}+{\tilde{z}}_{t}⊙\left({\hat{h}}_{t-1}-{\tilde{n}}_{t}\right)$.

This expression requires one final elementwise multiplication. The update gate, reset gate, and candidate polynomial are evaluated elementwise over all packed slots. Their coefficients are plaintext values, so polynomial coefficient multiplication uses plaintext ciphertext operations. Ciphertext multiplication is required only for forming powers, applying the reset gate, and combining the previous and candidate states.

### 4.4. Validation Locked Joint Calibration and Empirical Qualification

The Sigmoid and Tanh domains create a coupled decision because changing either polynomial changes the hidden states supplied to later gates. Calibration therefore evaluates complete pairs $\left(B_{\sigma },B_{tanh}\right)$ on complete recurrent trajectories with fixed network weights. The procedure separates model training, early stopping, candidate selection, and qualification. The split, candidate family, thresholds, and evaluation policy are fixed before the associated measurements. Section 6 specifies their experimental values and distinguishes the rules and evidence scope of each study.

The 49-pair grid used in the principal broad domain comparisons is $B_{\sigma }\in \{6,7,8,9,10,11,12\}$ and $B_{tanh}\in \{5,5.5,6,6.5,7,7.5,8\}$. Each pair is fitted by the least squares procedure in Section 4.3. For a complete polynomial trajectory i, let $M_{\sigma ,i}$ be the largest absolute update or reset preactivation and let $M_{tanh,i}$ be the largest absolute candidate preactivation. Define $S_{i}=max\;\left\{M_{\sigma ,i}/B_{\sigma },\;M_{tanh,i}/B_{tanh}\right\}.$

A finite trajectory respects both fitting domains when $S_{i}\leq 1$. A nonfinite trajectory receives an infinite score and is counted as an incorrect prediction. Each pair is also evaluated on a stress collection that repeats the transformed sequence twice without resetting the hidden state. The domain-constrained rule requires ordinary and stress finiteness for every checkpoint and a joint domain violation rate of at most 5% for each checkpoint.

Candidate selection combines these feasibility checks with a declared accuracy criterion. The assessment comparison in Section 6.12 maximizes mean macro accuracy among eligible pairs within two percentage points of the best finite candidate. Exact ties use ascending domain bounds. If no pair satisfies these requirements, the rule abstains. Accuracy selection without a domain threshold and direct error selection under the same accuracy budget serve as controls. The calibration experiment with Jacobian-based shortlist ranking is specified in Section 6.4 and Section 6.13. Its Jacobian measurements supply a sensitivity diagnostic and do not imply that this additional ranking improves the choice.

The selected pair is fixed before qualification. Let $S_{1},\ldots ,S_{n}$ be the scores on the qualification sample. At α=0.05, the one-based order statistic is $k=\left\lceil \left(n+1)(1-\alpha \right)\right\rceil .$

The empirical coverage check passes when k≤n and $q=S_{\left(k\right)}\leq 1$. Ordinary finiteness and matched stress finiteness are additional pass conditions. A task utility decision must also be supplied explicitly before the combined requirements can pass. Unspecified utility, nonfinite scores, and invalid trajectory status cannot be interpreted as successful qualification. The rank threshold alone cannot enforce these conditions.

For k≤n, under exchangeability of the qualification scores and a future score $S_{n+1}$, with the model and candidate fixed independently of the sample, the random threshold satisfies $Pr\;\left(S_{n+1}\leq q\right)\geq \frac{k}{n+1}\geq 1-\alpha .$

This is a marginal statement over the random sample and future trajectory. It does not provide a conditional population coverage guarantee after an observed pass. A fixed class allocation or dependence between archive rows can invalidate the intended exchangeability argument. Requiring several checkpoints to pass also does not by itself establish simultaneous coverage. The empirical check remains useful as a declared sample assessment without assigning it an unsupported population interpretation.

The roles of the thresholds are distinct. The 5% selection limit is a prespecified engineering budget on the fraction of trajectories with a normalized domain score above one. In qualification, α = 0.05 fixes the order-statistic rule before evaluation; the numerical threshold q is then obtained from the locked model’s qualification sample and is compared with the domain boundary one. Thus q is data-dependent, while the decision rule is fixed. The p95 of hidden-state RMSE in the results is a descriptive error statistic and does not determine this coverage threshold. Section 7.12 reports sensitivity of the engineering budgets.

A Wilks tolerance statement additionally specifies both population content and confidence, for example 95% content at 95% confidence, and uses a sample-size-dependent order statistic [4,20]. The marginal rank inequality above and the recorded empirical pass decision do not by themselves supply that two-level guarantee. Stating the score, sample size, rank and separate utility checks makes each reported qualification decision reproducible and its statistical interpretation explicit.

The lock records the protocol identity, checkpoint hashes, partition indices, candidate outcomes, coefficients, and qualification results. Test access requires a separate authorization bound to the locked model. A research comparison can authorize evaluation of every locked variant, including qualification failures, provided that no result changes the candidate and every outcome is retained. This separation controls the recorded information flow and prevents a qualification pass from being confused with either a test access permit or a deployment guarantee. Implementation provenance and differences between experimental rules are retained in Section 6.13.

### 4.5. Ciphertext Packing and Linear Transformations

The packed layout places one hidden coordinate across a contiguous block of samples. Let M denote the packed batch size and let m denote the hidden dimension. Coordinate j of sample b is stored at slot jM+b, where 0≤j<m and 0≤b<M. A full layout satisfies Mm=S, where S is the number of used slots. The intended conjugate invariant profile provides real slots, while the standard structural profile encodes real values in complex slots. Input feature vectors with dimension below m are padded with zeros before applying the input matrices. This convention allows the input and recurrent paths to use the same packed linear kernel.

Each plaintext weight matrix is encoded through cyclic diagonals. If ct(v) contains the packed input and $P_{k}$ contains the encoded k th diagonal, a packed matrix product can be expressed as

$$PCMM\;\left(ct(v),W\right)=\sum_{k=0}^{m-1}Rot\;\left(ct(v),kM\right)⊙P_{k}.$$

The diagonal products are reorganized with a baby-step giant-step (BSGS) schedule to reduce the number of distinct rotations [25]. Let $m_{2}=\lceil \sqrt{m}\rceil$ and $m_{1}=\lceil m/m_{2}\rceil$. The server precomputes baby rotations by multiples of M, evaluates groups of diagonal products, and then applies giant rotations by multiples of $m_{2}M$. The schedule requires approximately $m_{1}+m_{2}-2$ rotation steps, together with m plaintext multiplications and their additions. Independent giant step groups can be evaluated by separate worker threads.

One GRU time step evaluates six plaintext ciphertext matrix products. They correspond to the input and recurrent paths of the update gate, reset gate, and candidate state. The two paths of each gate are added at a common level, and the bias is encoded as a plaintext vector. The update and reset linear transformations can run independently. The input candidate path can also be prepared while the two gate polynomials are evaluated. This schedule exposes parallel work before the reset gate is multiplied with the recurrent candidate path.

### 4.6. OneBoot Encrypted Recurrent Step

At time t, the server receives a fresh encrypted input $ct({\tilde{x}}_{t})$ and a refreshed hidden state $ct({\hat{h}}_{t-1})$. It first evaluates the six packed matrix products. The update and reset affine outputs are passed through the locked $p_{\sigma ,B_{\sigma }^{⋆}}$. The recurrent candidate output is aligned with the reset gate level, multiplied elementwise by the reset gate, rescaled, and added to the input candidate output. The resulting ciphertext is passed through $p_{tanh,B_{tanh}^{⋆}}$.

The previous hidden state is aligned with the candidate level before the final update. The server subtracts the candidate from the previous state, multiplies the difference by the update gate, rescales the product, and adds the candidate. This operation produces $ct({\tilde{h}}_{t})$. Every dependency required for the current recurrent round has now been resolved.

The server applies

$$ct({\hat{h}}_{t})=Bts\;\left(ct({\tilde{h}}_{t})\right)$$

and uses the refreshed ciphertext as the only recurrent state of the following iteration. The refreshed state is also added to the appropriate temporal pooling accumulator. These accumulators receive values with a common scale and require no ciphertext multiplication during recurrence. One Bootstrapping operation therefore serves the complete GRU state transition and the subsequent pooling path.

The placement of Bootstrapping after the final hidden update is enabled by the single persistent state of the GRU. PPLSTM carries a cell state and a hidden state and refreshes both persistent values within its recurrent schedule [6]. OneBoot GRU completes all gate and state operations within the residual chain and refreshes one ciphertext at the round boundary. This placement preserves a simple level invariant because each iteration begins with the input and hidden state at their designated high levels.

### 4.7. Level Management

In the intended residual profile, fresh input and refreshed hidden-state ciphertexts start at level 10. The six packed affine paths consume one level and yield level-9 preactivations. The cubic update and reset gates consume two more levels and reach level 7. Multiplying the reset gate by the recurrent candidate path yields level 6 after alignment and rescaling. The degree-seven candidate polynomial reaches level 3, and final hidden-state interpolation reaches level 2. Thus, the measured path is 10 → 9 → 7 → 6 → 3 → 2. These levels describe the critical dependency path, not the sum of all multiplication counts in parallel gate branches.

The level-10 to level-2 transition consumes eight residual levels before one actual hidden-state refresh restores the configured output level and scale. The refresh input remains above the implementation’s recorded minimum input level. Repeating the same measured cycle establishes a level invariant for the tested circuit. It does not imply numerical contraction or identical precision for every future input; Section 5 and Section 7 measure those properties separately. The margin to the refresh boundary is material because another degree or scale choice can change whether the same parameters admit a further step.

The structural and intended profiles instantiate the same eight-level dependency pattern at different level offsets: the intended path is 10 → 9 → 7 → 6 → 3 → 2 → 10 after refresh, whereas structural closure is 9 → 8 → 6 → 5 → 2 → 1 → 9. In both cases, the final trained readout uses one additional level outside the recurrent loop. The degree probes in Section 7.11 additionally measure seven-, eight-, and nine-level cell variants, including the resulting margin to the refresh input. Table 5 separates ring size, security evidence, and measurement scope; a smaller arithmetic test profile does not inherit the security claim of the intended profile.

Level alignment uses explicit modulus dropping before additions or ciphertext multiplications. Rescaling follows every ciphertext product and every plaintext polynomial multiplication that raises the scale beyond the target. The encoded polynomial coefficients and matrix diagonals use scales chosen to return outputs near the common default scale. The circuit records the level after each major stage and verifies the decrypted value against a plaintext polynomial reference. This check separates arithmetic implementation error from the task error caused by replacing the exact activation functions.

### 4.8. Computational Properties

Computational cost has an offline calibration component and an online encrypted component. Let Kσ and Kτ be the numbers of Sigmoid and Tanh bounds, M the fitting-grid size, and pσ and pτ the numbers of fitted basis coefficients. Dense least squares costs O(Mp^2^ + p^3^) per interval. The implementation fits each distinct interval once and reuses its coefficients across the Cartesian product. Thus, seven bounds per activation require 14 scalar fits, while producing K = KσKτ = 49 joint candidates. The separate 200,001-point error audit is also performed once per fitted interval.

For S checkpoints, Nsel selection sequences of length T, input dimension d and hidden dimension m, the dense plaintext trajectory-search cost at fixed polynomial degrees is

(8) $$O\;(KSN_{sel}T(md+m^{2})).$$

Ordinary and doubled-length screening together process three times the natural horizon, without changing this asymptotic order. Stored checkpoint-specific exact references can be reused. Qualification evaluates only the locked pair in the calibration study; the separate prospective proxy comparison evaluates all 49 pairs and incurs the corresponding extra cost. Dense Jacobian formation, matrix products and spectral-norm evaluation have an additional cubic dependence on m. With Q shortlisted candidates, nJ sampled trajectories and a fixed set of window lengths, a direct implementation costs O(QSnJT m^3^) up to the window-dependent constant. These costs are plaintext development costs, not homomorphic operations.

Online, each step performs six packed matrix products, two cubic gates, a reset product, one degree-seven candidate, a final state product and one refresh. Under the padded diagonal layout, a matrix product uses m plaintext multiplications and approximately O(√m) rotations with baby-step giant-step scheduling [25]. The critical path consumes eight levels. Changing fitted domains preserves this symbolic depth because the degrees and evaluation graph are fixed; it does not establish equal numerical error for different coefficients.

For T steps and packed batch size B, an operation-cost model is T[6Cmat + 2Cσ + Cτ + 2Cmul + Cboot] + Creadout, where each C denotes the cost of the corresponding implemented primitive at the specified parameters. This is an accounting model, not a latency prediction: alignment, parallelism, key switching and memory traffic affect measured time. Dividing by B describes full-batch amortization only when distinct samples are actually packed. The replicated structural cases do not measure heterogeneous-batch throughput. One refresh per step assumes that the entire recurrent state fits the declared single-ciphertext layout; larger states may require multiple ciphertext refreshes.

The structural comparison with encrypted LSTM follows from persistent state and gate count. An LSTM has four affine gates and carries two recurrent states. A GRU has three affine gates and carries one recurrent state. PPLSTM also evaluates the inverse square root required by RMSNorm and refreshes its two persistent state ciphertexts [6]. OneBoot GRU evaluates six input and recurrent matrix paths and refreshes one hidden state. This reduction motivates a matched system comparison and does not determine the end-to-end speedup by itself.

The method preserves the noninteractive property of CKKS inference. The client encrypts the sequence and receives the encrypted prediction after server evaluation. All intermediate recurrent states, gate values, polynomial inputs, and pooled representations remain encrypted. The server uses public model weights and evaluation keys throughout the computation. The next section analyzes the error propagation and stability properties of this polynomial recurrent map.

### 4.9. Extension: Domain Constrained Polynomial Fine-Tuning

The core calibration experiment keeps weights fixed. The supplementary fine-tuning study asks whether a training objective can improve the trajectory generated inside fixed domains. It is evaluated separately and does not change the model assessed by the core calibration procedure. The extension fixes the pair at $B_{\sigma }=8$ and $B_{tanh}=6$, with the same least squares coefficients used by the selected model. All student forward passes use the deployed polynomial activations. The exact activation teacher remains fixed. Training uses public plaintext examples and requires no encrypted training operation.

For sequence i, gate path g∈{z,r,n}, and hidden coordinate j, define the full trajectory score

$$s_{i,g}(\theta )={max}_{1\leq t\leq T_{i},1\leq j\leq m}\frac{\left|a_{i,t,j}^{g}(\theta )\right|}{B_{g}},\;B_{z}=B_{r}=8,\;B_{n}=6.$$

The preactivations are evaluated on the student’s polynomial trajectory. This distinction is important because an exact teacher trajectory can remain bounded while its polynomial replacement diverges. The training penalty sums over the three path maxima, whereas the joint qualification score is their maximum. With a fixed margin ρ=0.9, the batch penalty is

$$R_{dom}(\theta )=\frac{1}{b}\sum_{i=1}^{b}\sum_{g\in \{z,r,n\}}{\left[max\left(0,s_{i,g}(\theta )-\rho \right)\right]}^{2}.$$

Absolute values, maxima, and the squared hinge are evaluated only during plaintext training. Automatic differentiation propagates through the selected maxima. No maximum, comparison, or clipping operator is added to encrypted inference. The margin encourages an interior buffer relative to the fitting domain, while the soft objective permits residual violations.

Let $u_{i}$ and $v_{i}$ denote the frozen teacher and polynomial student logits. With distillation temperature τ=2, define $q_{i}=softmax(u_{i}/\tau )$ and $p_{i}=softmax(v_{i}/\tau )$. The objective follows teacher distillation through softened predictions and is

$$L_{\lambda }=L_{CE,w}+0.5\;\tau^{2}\frac{1}{b}\sum_{i=1}^{b}D_{KL}(q_{i}\|p_{i})+\lambda \;R_{dom},$$

where $L_{CE,w}$ is the class-weighted cross-entropy used by the implementation. The ordinary control uses λ=0, and the constrained arm uses λ=10. Both arms update all student parameters using Adam with learning rate 0.0003, weight decay 0.0001, batch size 64, and gradient norm clipping at one. They run twelve fixed epochs from matched initialization and batch order. The final epoch is retained, with no checkpoint selection from qualification or test performance.

The original paired study compares λ = 0 with λ = 10 and cannot identify an optimal penalty weight. The revision-stage sensitivity study in Section 6.14 and Section 7.9 evaluates a wider fixed grid with matched initialization and optimization. These additional runs assess parameter dependence on reused development data, without changing any original model-selection or test result.

A nonfinite loss or gradient terminates the run and is recorded as a failed outcome. The implementation does not silently clip hidden states, replace seeds, or retry with modified hyperparameters. Training evaluates ordinary length trajectories. Doubled-length trajectories are reserved for assessment in this experiment, so the objective supplies no direct training constraint on the extended horizon.

This operation changes the model weights without changing the cell’s symbolic multiplicative depth or refresh placement. It leaves numerical encrypted precision as a separate validation requirement because weight magnitudes and visited states can change. A new checkpoint must be locked and evaluated under the qualification and research access rules described in Section 6. The original encrypted trace cannot serve as a measured trace for the fine-tuned model.

## 5. Stability Analysis

Polynomial activations create a numerical question distinct from ciphertext depth. This section separates activation replacement, CKKS arithmetic perturbations and recurrent amplification. The complete derivative is evaluated for the reset-after-linear cell defined in Section 3.2. Following recurrent sensitivity analyses [21,22], local Jacobian norms and ordered products are used as diagnostics. Finite sampled trajectories, a conservative norm bound and a global contraction certificate are different statements; the experiments support only the first two within their specified scope.

### 5.1. Stability Setting and Error Sources

Let $F_{t}^{ex}$ denote the GRU transition at time t with exact Sigmoid and Tanh functions. Let $F_{t}^{pol}$ denote the corresponding transition with the jointly selected degree 3 Sigmoid polynomial and degree 7 Tanh polynomial. The subscript t reflects the dependence of each map on the current input ${\tilde{x}}_{t}$. Starting from a common initial state, the exact and polynomial plaintext trajectories satisfy

$$h_{t}^{ex}=F_{t}^{ex}\;\left(h_{t-1}^{ex}\right)$$

$$and\;h_{t}^{pol}=F_{t}^{pol}\;\left(h_{t-1}^{pol}\right).$$

The decrypted state produced by CKKS is represented as

$$h_{t}^{ckks}=F_{t}^{pol}\;\left(h_{t-1}^{ckks}\right)+\xi_{t},$$

where $\xi_{t}$ collects encoding error, rescaling error, key switching error, polynomial evaluation error caused by finite CKKS precision, and the approximation error introduced by Bootstrapping. This representation treats the cryptographic computation as a perturbation of the polynomial plaintext map after decryption. It does not require each arithmetic error to be independent.

The difference between the exact and polynomial maps is the activation replacement error. Its size depends on the preactivations reached by each trajectory, while CKKS adds a further arithmetic perturbation [1,5]. Domain screening measures whether evaluated preactivations leave the fitted intervals. Neither input clipping nor a successful sample screen proves that future states remain within those intervals. The following bound therefore states its Lipschitz assumption explicitly rather than deriving it from observed coverage.

### 5.2. Recurrent Perturbation Bound

Define the total encrypted inference error as

$$e_{t}=h_{t}^{ckks}-h_{t}^{ex}.$$

At the exact state, define the activation replacement error

$$\alpha_{t}={\left\|F_{t}^{pol}\;\left(h_{t-1}^{ex}\right)-F_{t}^{ex}\;\left(h_{t-1}^{ex}\right)\right\|}_{2}$$

and let $\epsilon_{t}=\|\xi_{t}{\|}_{2}$. Suppose that $F_{t}^{pol}$ is Lipschitz continuous with factor $L_{t}$ on the line segment connecting $h_{t-1}^{ex}$ and $h_{t-1}^{ckks}$. Adding and subtracting the polynomial transition at the exact state gives

$$\|e_{t}{\|}_{2}\leq L_{t}\|e_{t-1}{\|}_{2}+\alpha_{t}+\epsilon_{t}.$$

Repeated application yields

$$\|e_{T}{\|}_{2}\leq (\prod_{j=1}^{T}L_{j})\|e_{0}{\|}_{2}+\sum_{k=1}^{T}(\prod_{j=k+1}^{T}L_{j})(\alpha_{k}+\epsilon_{k}),$$

where an empty product equals one. This expression makes the interaction between approximation quality and recurrent dynamics explicit. Small per-step errors are sufficient only when the subsequent transition products remain controlled. If $L_{t}\leq \rho <1$ and $\alpha_{t}+\epsilon_{t}\leq \delta$ over an invariant domain, then

$$\|e_{T}{\|}_{2}\leq \rho^{T}\|e_{0}{\|}_{2}+\delta \frac{1-\rho^{T}}{1-\rho }.$$

The second term approaches the finite error floor δ/(1−ρ). This uniform contraction condition is sufficient and can be conservative. Time-varying recurrent systems can attenuate perturbations over several steps even when some individual transitions have a local factor above one. Products of recurrent Jacobians therefore provide a more informative empirical diagnostic than a single recurrent weight norm or an isolated activation approximation error. Similar Jacobian products govern gradient growth and decay in recurrent networks [21].

### 5.3. Complete Polynomial GRU Jacobian

Fix one input and suppress the time index. With the selected polynomial coefficients, define

(9) $$a_{z}=W_{z}\tilde{x}+U_{z}h+b_{z},\;z=p_{\sigma }(a_{z}),$$

(10) $$a_{r}=W_{r}\tilde{x}+U_{r}h+b_{r},\;r=p_{\sigma }(a_{r}),$$

(11) $$v=U_{n}h,\;a_{n}=W_{n}\tilde{x}+r⊙v+b_{n},\;n=p_{\tau }(a_{n}).$$

Here pτ denotes the selected Tanh polynomial, D(q) is the diagonal matrix with diagonal q, and a prime denotes the scalar derivative evaluated element-wise. The update and reset derivatives with respect to h are

(12) $$J_{z}=D(p_{\sigma }^{\prime }(a_{z}))U_{z},\;J_{r}=D(p_{\sigma }^{\prime }(a_{r}))U_{r}.$$

The candidate has a direct path through U_n_h and an indirect path through the reset gate. The product and chain rules give

(13) $$\frac{\partial a_{n}}{\partial h}=D(r)U_{n}+D(v)J_{r},$$

(14) $$J_{n}=D(p_{\tau }^{\prime }(a_{n}))[D(r)U_{n}+D(v)J_{r}].$$

For F(h) = n + z ⊙ (h − n), differentiation gives

(15) $$J_{F}=D(z)+D(1-z)J_{n}+D(h-n)J_{z}.$$

These terms respectively describe direct state retention, the full candidate path and update-gate sensitivity. In particular, D(v)Jr cannot be discarded: it is the reset gate’s dependence on the previous state. A bound on a recurrent weight matrix alone does not bound JF, because gate values, activation derivatives and the current state also enter the expression.

For the cubic Sigmoid fit, the derivative is

(16) $$p_{\sigma }^{\prime }(u)=a_{1}+3a_{3}u^{2}.$$

The locked TwoPatterns fit on [−8, 8] has a_1_ = 0.150115055220 and a_3_ = −0.001592822068, giving

(17) $$p_{\sigma }^{\prime }(u)=0.150115055220-0.004778466204u^{2}.$$

Writing the odd degree-seven Tanh fit as pτ(u) = c_1_u + c_3_u^3^ + c_5_u^5^ + c_7_u^7^ avoids confusing its coefficients with those of the Sigmoid fit. Its derivative is

(18) $$p_{\tau }^{\prime }(u)=c_{1}+3c_{3}u^{2}+5c_{5}u^{4}+7c_{7}u^{6}.$$

These expressions allow the Jacobian to be evaluated directly from the model parameters and the preactivations collected along a trajectory. The implementation passes each selected candidate coefficient vector to a generic polynomial derivative and therefore uses the sig8 coefficients in the frozen stability ranking. A post-lock recomputation produced the same five checkpoint statistics as the lock with a maximum absolute difference of zero. The analytical implementation was also compared with automatic differentiation on a random three-dimensional cell. The maximum absolute difference between corresponding Jacobian entries was $2.98\times 10^{-8}$, which verifies the implemented derivative to single-precision accuracy.

### 5.4. State Dependent Norm Bound

For the Euclidean induced matrix norm, ‖D(q)‖_2_ = ‖q‖∞. Applying the triangle inequality and submultiplicativity to the three terms of JF in Equation (15) first gives

(19) $$\|J_{F}{\|}_{2}\leq \|z{\|}_{\infty }+\|1-z{\|}_{\infty }\|J_{n}{\|}_{2}+\|h-n{\|}_{\infty }\|J_{z}{\|}_{2}.$$

Define the state-dependent derivative factors

(20) $$d_{z}=\|p_{\sigma }^{\prime }(a_{z}){\|}_{\infty },\;d_{r}=\|p_{\sigma }^{\prime }(a_{r}){\|}_{\infty },\;d_{n}=\|p_{\tau }^{\prime }(a_{n}){\|}_{\infty }.$$

Then ‖Jz‖_2_ ≤ dz‖Uz‖_2_ and ‖Jr‖_2_ ≤ dr‖Ur‖_2_. Substitution into the candidate derivative gives ‖Jn‖_2_ ≤ βn, where

(21) $$\beta_{n}=d_{n}(\|r{\|}_{\infty }\|U_{n}{\|}_{2}+\|v{\|}_{\infty }d_{r}\|U_{r}{\|}_{2}).$$

Consequently, a computable upper bound is ‖JF‖_2_ ≤ B(h, x), where

(22) $$B(h,x)=\|z{\|}_{\infty }+\|1-z{\|}_{\infty }\beta_{n}+\|h-n{\|}_{\infty }d_{z}\|U_{z}{\|}_{2}.$$

Every factor is evaluated at the same state and input, using the locked coefficients and recurrent matrices. To obtain the Lipschitz factor required in Section 5.2, the bound must hold along the whole line segment between the compared states; its value at one endpoint is insufficient. A global contraction argument additionally needs a forward-invariant domain and a uniform supremum below one. Sampled maxima supply neither condition.

Domain containment also does not force polynomial gate values into [0, 1]. Using the rounded Sigmoid coefficients above, the stationary points inside [−8, 8] occur near u = ±5.60490. The fitted polynomial takes values approximately 1.06092 and −0.06092 at these points. These are analytical evaluations of the stated polynomial, not observed data frequencies. They show why an in-domain state update need not be a convex combination and why the gate-value factors in B(h, x) cannot simply be replaced by one. Wider fitting domains may change both values and derivatives; improvement in coverage does not imply improvement in this bound.

### 5.5. Local and Multistep Dynamics

Let $J_{t}$ be the complete Jacobian evaluated at the hidden state reached at time t. For a window of k recurrent steps, define

$$P_{t,k}=J_{t}J_{t-1}\cdots J_{t-k+1}.$$

The spectral norm $\|J_{t}{\|}_{2}$ measures local sensitivity at one state. The norm $\|P_{t,k}{\|}_{2}$ measures first-order sensitivity across the entire window. The ordering is important because the GRU is a time-varying nonlinear system whose gates change with both the input and the state. Occasional local expansion can be followed by stronger contraction in later transitions. Consequently, a local norm above one does not establish trajectory divergence, and a small average of local norms does not establish multistep contraction. Directly evaluating the ordered product preserves the interaction among successive Jacobian directions.

The bound in Section 5.4 identifies the quantities needed for a uniform contraction argument. The empirical studies instead measure complete trajectories, sampled Jacobian products, and encrypted arithmetic error directly. The expanded candidate analysis in Section 7.13 tests sensitivity as a predictor across 49 domain pairs on two tasks, while Section 7.10 and Section 7.16 examine short target-profile and full structural-profile encrypted recurrence.

### 5.6. Empirical Jacobian Diagnostics

The frozen protocol evaluates the full Jacobian only for candidates in the accuracy shortlist on a deterministic subsample of at most 32 candidate selection trajectories per checkpoint. Ordered products use horizons of 8, 16, and 32. Each trajectory contributes the largest base-10 logarithm of a product spectral norm over the registered horizons. The checkpoint statistic is the empirical 95th percentile of these trajectory values, and the candidate risk is the largest statistic across the five checkpoints. Nonfinite Jacobians or products make the candidate ineligible for stability ranking. The successful TwoPatterns run has one shortlisted candidate. Its Jacobian values therefore provide diagnostics and do not demonstrate an improvement in candidate selection.

For the locked TwoPatterns pair sig8_tanh6, the five checkpoint statistics are 0.9444, 0.9270, 0.7183, 0.6691, and 0.9021. These logarithmic values correspond to observed product norms above one on some registered windows. The selected model therefore does not satisfy empirical multistep contraction across all sampled segments. The statistic describes sampled sensitivity and cannot be interpreted as a stability certificate or evidence of an incremental ranking benefit.

The original small-cell automatic-differentiation check supports the analytical implementation. A separate revision-stage check uses the actual five locked TwoPatterns models in float64 and the first 32 recorded selection trajectories per checkpoint. It evaluates all 128 steps, giving 4096 state-input pairs per checkpoint and 20,480 in total. At each pair, the spectral norm of the complete Jacobian is compared with B(h, x). Automatic differentiation additionally checks the first trajectory at steps 1, 64, and 128 of every checkpoint. This diagnostic reuses development data and does not select or qualify a new model. Table 3 reports the measured Jacobian norms and corresponding bounds for each seed.

The bound is not violated at any of the 20,480 sampled states (numerical comparison tolerance 10^−10^). Checkpoint p95 actual norms are 1.197–1.243, whereas p95 bounds are 4.001–4.578; median bound-to-norm ratios are 2.383–2.547. The maximum discrepancy from automatic differentiation over the 15 real-model checks is 2.22 × 10^−16^. These results verify the formula and quantify its conservatism. They do not establish uniform contraction: sampled local norms exceed one, and the original multistep diagnostics also contain expanding windows. The per-seed p95 values are higher-order statistics over the 4096 state-input pairs of each checkpoint; they are distinct from the trajectory-level product statistic above.

### 5.7. Long-Horizon Trajectory Tests

Every candidate is evaluated on natural selection trajectories and deterministic doubled-length repetitions. TwoPatterns sig8_tanh6 has zero nonfinite trajectories in both collections across all five checkpoints. Its qualification scores meet the empirical rank threshold. The historical calibration rule and the stronger prospective qualification rule are distinguished in Section 6.13. On the official test set, all polynomial trajectories remain finite, and the mean joint domain violation rate is 0.830%. These observations do not guarantee bounded hidden states under arbitrary clipped inputs.

Wafer and ElectricDevices make the distinction between coverage and dynamics inspectable. Their original domain filters abstain before official testing. The new paired gate interventions in Section 7.15 identify the candidate-state activation as a useful control point: exact candidate activation preserves finiteness throughout all tested Wafer settings and the middle and widest ElectricDevices settings. These experiments explain observed trajectories without changing the original selection decisions.

The encrypted evidence includes the original 64-step and four 128-step cases, twelve additional distinct 128-step sequences, and short end-to-end execution using the intended security-target profile. The twelve added full sequences all agree with their plaintext polynomial predictions; their peak hidden-state and output-logit errors are characterized as distributions in Section 7.16. The long-sequence experiments use the structural profile and complement the separate target-profile validation in Section 7.10.

### 5.8. Scope of the Stability Claim

The stability evidence consists of finite evaluated trajectories, measured coverage and fidelity, verified complete Jacobians, and successful encrypted arithmetic under stated parameters. The candidate study clarifies that Jacobians characterize perturbation propagation, whereas observed polynomial error also includes the approximation defect injected at each step. Direct trajectory error and domain screening therefore complement sensitivity analysis. All qualification decisions are empirical; a global certificate would require the additional conditions below.

A global certificate would require a forward invariant hidden state set derived from the clipped input domain, certified intervals for all three preactivations, and uniform bounds for the polynomial values and derivatives. Substitution of these quantities into the state-dependent bound would need to produce a factor below one or another finite-horizon bound suitable for the application. Training could include a penalty on the complete Jacobian, explicit state projection, or a homomorphically compatible bounded state mechanism. The per-step CKKS and Bootstrapping error bound would then enter the perturbation term δ. The present work keeps this certification path distinct from its empirical qualification claim.

### 5.9. Stability Boundaries of the Fine-Tuning Extension

The separately reported fine-tuning extension does not alter the core stability claim. The Jacobian expressions remain valid after fine-tuning when evaluated with the updated weights and polynomial trajectory. Previously measured product norms remain measurements of the original checkpoints. They cannot be transferred numerically to the new weights. The current fine-tuning loss penalizes preactivation maxima and contains no Jacobian product term.

Reducing preactivation excursions can keep more evaluated states within the region where the polynomial approximation error was measured. This provides a mechanism for improved domain control. It does not impose a uniform bound on the recurrent matrices, gate values, or Jacobian products. Even zero penalty on every training trajectory would establish only a sampled property. A forward invariant hidden set does not follow from a finite collection of inactive penalties.

The transfer outcomes expose two distinct limitations. Some initial polynomial trajectories are nonfinite before optimization, which prevents ordinary gradient-based fine-tuning from obtaining a meaningful loss. Another completed checkpoint has finite ordinary qualification trajectories but nonfinite doubled length trajectories. The first case concerns the feasibility of starting the repair procedure, while the second concerns the horizon over which the repaired model remains numerically useful. Neither limitation is resolved by restoring ciphertext levels.

## 6. Experimental Methodology

The evaluation asks whether a fixed-degree polynomial GRU satisfies its range, trajectory, and task requirements, and whether its computation can be realized with repeated encrypted refresh. It combines calibration, direct comparisons of selection criteria, complete arithmetic checks, and a separately evaluated fine-tuning extension. Frozen test evaluations and retrospective diagnostics retain distinct roles. The rules and evidence scope of each study are summarized in Section 6.13.

### 6.1. Research Questions

The experiments address four linked questions. First, does a domain constraint change the chosen polynomial pair beyond accuracy and finite-trajectory screening? Same-checkpoint, same-grid selector comparisons isolate that decision. Second, do coverage and sampled Jacobian diagnostics predict direct recurrent fidelity? Paired exact-versus-polynomial trajectories and candidate correlations evaluate this question. Third, do the selected models retain task utility beyond selection data? Locked qualification and once-only official-test results provide the stated sample evidence. Fourth, can CKKS execute the trained recurrent circuit with one refresh per step? Structural full-sequence runs, and separate short target-profile runs compare decrypted results with the same plaintext polynomial circuit. A matched GRU–LSTM implementation measures the cost of the respective recurrent graphs under common parameters. Ciphertext-level restoration alone answers none of the numerical or task questions.

The protocol choices follow these questions. Reusing each checkpoint across the 49 candidate pairs isolates the effect of polynomial coefficients and domain bounds; ordinary and doubled-length trajectories expose both immediate and feedback-driven failures. A disjoint qualification partition assesses the locked choice, while official tests are accessed according to the recorded study policy. Repeated inputs are deliberately a stress case, not a model of all naturally occurring long sequences. The four-step target-profile test checks the executable secure-parameter graph; the 128-step structural tests expand numerical coverage at feasible cost. Because these experiments use different parameter profiles and sampling units, their conclusions are reported separately in the corresponding table.

### 6.2. Dataset Roles and Access Control

As shown in Table 4, each dataset has a declared experimental role and test-access rule [26]. Model fitting, selection, and qualification use separate subsets of each official training partition. Candidate and checkpoint identities are locked before qualification outcomes are used. Reused selection or qualification observations are labeled retrospective and cannot redefine an already reported test result.

### 6.3. Input Processing and Model Architecture

Every raw value is clipped to [−4,4]. Causal differences with lags 1, 2, 4, 8, and 16 are computed and clipped to the same interval. For a univariate series, the transformed input has dimension six. The classifier contains one GRU layer with hidden dimension 32, an eight-bin temporal pyramid mean, and a linear output head. Exact Sigmoid and Tanh activations are used during training. Preprocessing is public and deterministic, and no statistics are recomputed from an official test partition.

Adam uses a learning rate $10^{-3}$, weight decay $10^{-4}$, batch size 64, and gradient-norm clipping at 1. Training lasts at most 50 epochs with early-stopping patience 10. The fixed seeds are 11, 22, 33, 44, and 55. Inverse class-frequency weighting follows the frozen classification pipeline. All checkpoints are trained before polynomial candidate selection.

### 6.4. Calibration Study Design

The calibration study stratifies the official training split with PCG64 seed 20260902. Within each class, downward rounding assigns 65% of examples to model training, 15% to early stopping, and 10% to candidate selection. The remainder forms qualification. The protocol fails if any class cannot populate every subset.

Sigmoid candidates are degree 3 with basis $1,x,x^{3}$ and constant coefficient 0.5. Tanh candidates are degree 7 with basis $x,x^{3},x^{5},x^{7}$. Coefficients use unweighted double-precision least squares on 20,001 uniform points, and maximum scalar error is evaluated on 200,001 points. The smaller grid uses bounds 3 to 6 in increments of 0.5 for both functions. The broader grid uses Sigmoid bounds 6 to 12 in increments of 1 and Tanh bounds 5 to 8 in increments of 0.5. Each grid contains 49 joint candidates.

Every candidate is evaluated on complete selection trajectories and deterministic trajectories that repeat the sequence twice without resetting the hidden state. Eligibility requires finite ordinary and stress trajectories for every seed and a joint domain violation rate no greater than 5% for any seed. Candidates within 0.5 percentage points of the best mean macro accuracy enter stability ranking. Complete Jacobian products use horizons 8, 16, and 32 on at most 32 deterministic trajectories. Ties are resolved by maximum checkpoint stability statistic, final hidden root mean square error (RMSE), scalar approximation error, and lexicographic bounds.

### 6.5. Qualification and Research Test Evaluation

For each checkpoint, normalized scores are ordered and k=⌈(n+1)(1−0.05)⌉ is selected. Coverage requires k≤n and $S_{\left(k\right)}\leq 1$. Ordinary finiteness, matched stress finiteness, and a prespecified utility condition are additional requirements. A failure does not authorize a different candidate from the same qualification sample. The decision is empirical unless the assumptions needed for a population interpretation are established.

The prospective reliability study applies this complete rule and requires qualification macro accuracy within five percentage points of the same checkpoint’s exact model. The calibration study uses the rank gate specified in Section 6.13. Each study has a separate policy determining whether rejection terminates official testing or remains a reportable arm in a locked research comparison. The dataset lock records protocol identity, partition indices, checkpoint hashes, candidate records, selected coefficients, and qualification observations. Test records include exact and polynomial macro accuracy, their difference, nonfinite counts, and domain violations for every scheduled seed. Test outcomes cannot change the model.

### 6.6. Post Lock Ablation

The exploratory ablation uses only TwoPatterns selection and marginal-coverage qualification subsets. It compares fixed reference domains sig4_tanh3, one-axis Tanh and Sigmoid families, the frozen joint pair sig8_tanh6, and the widest broader-grid pair sig12_tanh8. Single-axis families use the frozen eligibility rule. If no candidate is eligible, the best diagnostic candidate is reported. The analysis does not reopen the official test set or alter the locked result.

A same-grid replay applies four fixed selection rules to the same 49 candidate aggregates. The rules maximize mean selection macro accuracy over the full grid, ordinary finite candidates, ordinary and stress finite candidates, and candidates also meeting the original domain threshold. Ties use hidden-state RMSE and candidate identifier. This retrospective comparison adds no forward passes, test reads, or qualification decisions. It isolates the effect of recorded filters and does not estimate runtime or population benefit.

### 6.7. Encrypted Real Model Closure

The encrypted closure uses the seed 11 TwoPatterns checkpoint, its trained weights, and sig8_tanh6 coefficients. The input is source index 70 from the training-derived marginal-coverage qualification subset, with the first 64 steps exported before encryption. Each packed ciphertext replicates the same state for the existing diagonal matrix kernel, so heterogeneous sample parallelism is not measured.

The implementation uses Lattigo v6.1.1 and a standard-ring CKKS structural profile with logN=12, 2048 real-populated complex slots, and maximum level 9 [27]. This reduced ring is intentionally outside the security target. Each step evaluates six trained affine paths, the locked degree 3 and degree 7 polynomials, the reset multiplication, and final hidden interpolation. State is decrypted only for verification before and after one actual Bootstrapping call. The refreshed output becomes the next input. Recorded metrics include levels, component latency, RMSE against a plaintext polynomial reference, maximum absolute error, finiteness, and Go heap usage.

The full arithmetic check evaluates 128-step sequences at source indices 70 and 533 crossed with seeds 11 and 55. These cases reuse qualification data and provide no new task-accuracy sample. The extended runner retains eight encrypted temporal-bin sums and incorporates each public reciprocal bin length into the trained head. The recurrent cycle is 9→1→9, and the readout consumes one additional level without another refresh. Intermediate decryption never feeds encrypted computation. Two Go tests compare the standard ring and encrypted readout with direct references.

### 6.8. Intended Parameter Profiles

Table 5 distinguishes the structural and intended profiles. On the approximately 16 GiB host, complete target-profile evaluation keys are now allocated by generating unique refresh rotation keys, limiting ring-conversion keys to the required residual level, and encoding matrices sequentially. These implementation changes preserve ring dimensions, modulus chains and secret distributions. Target-profile recurrence, refresh, output-head precision, key size and process memory are directly measured in Section 7.10.

For the intended profiles, the security audit fixes the Lattigo v6.1.1 source and parameter hashes, the conjugate-invariant residual ring at log N = 15 with log QP = 638, and the standard Bootstrapping ring at log N = 16 with log QP = 1520. Both use a sparse ternary main secret of Hamming weight 192; the Bootstrapping configuration uses an ephemeral secret of weight 32. The implementation’s default discrete-Gaussian error has standard deviation 3.2 and bound 19.2. The respective total moduli are 130 and 30 bits below the pinned sparse-secret reference limits documented by the audit. Uniform-ternary guideline limits are reported only as a secondary cross-check because they assume a different secret distribution [28]. This establishes version-pinned parameter-reference conformance for the target profile; no independent lattice-estimator run or attack-by-attack estimate for the exact tuple is claimed. The log N = 12 structural profile is excluded from this security conclusion.

### 6.9. Evidence Labels and Reporting Rules

Frozen official-test evaluations, retrospective replays and exploratory extensions retain separate labels. The original and expanded 128-step checks use the intentionally insecure structural ring. The four-step end-to-end and matched recurrent-baseline experiments use the intended 128-bit-target parameter profile with actual refresh. Their measured latency and memory apply to that configuration and scheduling policy. The LSTM baseline is a standard polynomial implementation sharing our kernels, rather than a reproduced PPLSTM system.

Accuracy is summarized by mean ± sample standard deviation across five training seeds where available; seeds sharing one dataset split are not independent population samples. Each encrypted sequence, rather than its replicated packed slots or dependent time steps, is the unit for sequence-level error distributions. System repeats use medians and observed ranges. Qualification-rank decisions are empirical because exchangeability with future observations is not established for these fixed archive partitions. Nonfinite outcomes remain in the denominators. The reported cryptographic conclusion is conformance to pinned parameter references; the descriptive sequence-error distributions and selected examples do not yield population-wide precision or stability guarantees.

To assess recurrent step-count sensitivity with the frozen model, we reused the twelve selected TwoPatterns qualification sequences, the seed-11 checkpoint, and the locked sig8_tanh6 coefficients. We summarized steps 32, 64, 96, and 128 from each archived 128-step structural-profile encrypted run; prefixes of one run remain dependent observations. At each horizon, we recorded finiteness, the ciphertext-level cycle, and the within-prefix peak hidden-state RMSE and coordinate error against the same plaintext polynomial circuit. The prefix analysis did not generate new encrypted readouts or predictions.

Separately, with the same inputs and weights, float64 polynomial and exact-activation rollouts were compared at 64 and 128 steps and at 256 steps obtained by repeating each 128-step input without resetting the state. We checked finiteness, frozen fitting-domain coverage, and each sequence’s peak hidden-state RMSE. The 256-step replay is a repeated-input stress test. Longer-step encrypted evidence uses the intentionally insecure structural profile (log N = 12); the target 128-bit profile was validated for four steps.

### 6.10. Reproducibility

The local experiment package contains executable protocols, Secure Hash Algorithm 256-bit (SHA-256) manifests, dataset partitions, model checkpoints, coefficient records, failure audits, per-seed metrics, encrypted traces and aggregation scripts. Tests cover deterministic splitting, polynomial fitting, joint scores, qualification order statistics and the test-access gate. The Go runner consumes an exported JSON artifact containing checkpoint and selection-lock hashes. The revision-stage sensitivity and norm checks have their own design record, source hash, per-seed outputs and protected-file verification; they do not overwrite the original experimental locks. Reproduction requires these supporting artifacts as well as the public University of California, Riverside (UCR) datasets [26].

### 6.11. Secondary Fine-Tuning Experiment

The extension compares ordinary and domain-constrained fine-tuning with fixed sig8_tanh6 coefficients, the same teacher, classification and distillation losses, batch sequence, and twelve-epoch budget. Only the constrained arm adds the trajectory maximum penalty from Section 4.9. The TwoPatterns pilot reuses development-informed subsets and forbids official test access.

Before acquiring PhalangesOutlinesCorrect and UWaveGestureLibraryX, the transfer recipe reserves a simple random 20% of each training set and stratifies the remainder. Five exact teachers initialize both arms. Models and failures are locked before qualification or official testing. A separate research policy evaluates all locked variants without deployment approval. The descriptive benefit criterion requires five completed seeds, mean accuracy within one percentage point of ordinary control, lower violations when the control has violations, and no increase in nonfinite trajectories. All failures remain in the comparison.

The additional UWaveGestureLibraryX diagnostic replays the two locked seed-55 fine-tuned checkpoints on all 3582 previously evaluated test sequences. For each checkpoint, the exact-activation reference uses the same fine-tuned weights; the trajectory metric is the peak hidden-state RMSE defined in Section 6.12. We report its median, higher-order-statistic p95 and maximum at 315 steps and at 630 steps obtained by repeating the transformed input without resetting state. Recurrence remains float32 and error accumulation uses float64. The other completed seed, 44, is retained as a paired consistency check, together with all original training statuses. This is a retrospective diagnostic with fixed models and coefficients.

### 6.12. Prospective Domain and Error Comparison

The reliability comparison fixes two datasets and five seeds before training-data acquisition. SyntheticControl has 300 training and 300 test sequences of length 60 in six classes. ChlorineConcentration has 467 training and 3840 test sequences of length 166 in three classes. Both are simulated archive tasks selected from public metadata and local feasibility, so they do not represent independent real application populations.

Using PCG64 seed 20260904, an unstratified permutation reserves 20% of training rows for qualification. The remainder is allocated within class to training, early stopping, and selection at fractions 0.625, 0.1875, and the remainder. Every class must occur in each partition. Counts are 147, 42, 51, and 60 for SyntheticControl and 233, 69, 72, and 93 for ChlorineConcentration. No fine-tuning is applied.

All selectors use identical checkpoints and the broader 49-pair grid. Ordinary and doubled-horizon finiteness are common requirements. Accuracy selection maximizes mean macro accuracy across seeds. Domain selection adds a worst-checkpoint violation limit of 5% and a two-point accuracy budget. Direct error selection minimizes mean checkpoint tail error under the same budget. Ties use ascending Sigmoid and Tanh bounds. An empty eligible set produces abstention. All selections are locked before qualification evaluation.

For trajectory i, the peak state error $E_{i}$ is the maximum hidden-state RMSE over time between polynomial and exact activations of the same checkpoint

$$E_{i}={max}_{1\leq t\leq T_{i}}\sqrt{\frac{1}{m}\sum_{j=1}^{m}{\left({\tilde{h}}_{i,t,j}-h_{i,t,j}\right)}^{2}}.$$

Here $T_{i}$ is trajectory length and m is hidden dimension. Each checkpoint contributes the 95th sample percentile using the higher order statistic without interpolation. Reported means average the five checkpoint quantiles. Nonfinite trajectories receive infinite error and count as incorrect predictions. Doubled trajectories repeat transformed features without resetting state. Logit error, absolute activation error, and the fraction with $E_{i}>0.5$ are secondary diagnostics.

The proxy comparison computes within-dataset, within-seed Spearman correlations across the 49 candidates. Its predictor is selection domain violation rate, and its outcome is qualification tail error. Constant predictors are not estimable. The descriptive criterion requires a correlation of at least 0.5 in four of five seeds on both tasks. The selection-benefit criterion requires at least 10% lower mean test tail error, nonincreased error in at least four seeds, macro accuracy within two points of accuracy selection and five points of the exact model, and no additional ordinary or stress nonfinite trajectories. These are descriptive criteria, not significance or equivalence tests.

All candidates receive qualification evaluation for the proxy comparison. Only locked nonabstaining candidates receive official test evaluation, regardless of qualification outcome. Duplicate selected pairs share measurements. Qualification uses the full checks in Section 6.5 and cannot change the selected pair. Weak models and unfavorable results remain included. A separately labeled retrospective TwoPatterns diagnostic uses fixed existing weights and previously used training-derived partitions without reading the official test set.

### 6.13. Experimental Protocols and Evidence Scope

The calibration study uses the stratified split and Jacobian shortlist rule of Section 6.4. Eligibility requires finite ordinary and doubled trajectories and compliance with the domain threshold. Its qualification rule applies the rank threshold and records nonfinite counts without a separate finite-trajectory rejection condition. The prospective study instead applies separate rank, ordinary-finiteness, stress-finiteness, and utility checks. It evaluates every locked nonabstaining selection under its research policy, including qualification failures, without changing selected models or omitting unfavorable outcomes. Both studies produce empirical pass decisions unless their population assumptions are justified.

The fine-tuning study retains every attempted seed and separates its development-informed TwoPatterns pilot from prospective transfer tasks. Qualification and encrypted arithmetic evidence do not transfer automatically to adapted weights. The same-grid replay and TwoPatterns diagnostic reuse recorded observations and supply no independent confirmation. Encrypted checks establish arithmetic execution for fixed cases under the profiles in Table 5. Table 6 summarizes the qualification and test-access rules for the recorded studies.

Interpretation follows the comparison design. Same-grid replays identify which filter changed a recorded choice but reuse development observations. Exact-versus-polynomial state traces measure activation-replacement error, whereas CKKS-versus-polynomial traces measure arithmetic error. The twelve encrypted full-sequence inputs were selected to span observed difficulty and therefore support descriptive error curves. Five seeds on a shared archive split reflect training variability, not independent population replication. These distinctions prevent an empirical pass or measured runtime advantage from being extended beyond the model, data, and parameter configuration actually tested.

### 6.14. Revision-Stage Sensitivity and Bound Verification

The additional study fixes λ ∈ {0, 0.1, 1, 10, 100} and seeds 11, 22, 33, 44 and 55 before its runs. It uses the original TwoPatterns exact teachers, sig8_tanh6 coefficients, 649 training rows and the same twelve-epoch optimization settings as Section 4.9. Preprocessing is loaded from the original frozen configuration, including the input clipping bound C = 4. Before fine-tuning, all five original qualification score vectors are reproduced with maximum absolute difference zero. Each arm restarts from its seed’s teacher and uses the same shuffled batches. The final epoch is retained, with no parameter or checkpoint selection from evaluation outcomes. A design record and source hashes identify this separate revision experiment.

After training, each completed arm is evaluated on the original 99 selection rows and 104 qualification rows, including doubled-length repetitions without resetting the state. Because these partitions were used in earlier development, all new outcomes are exploratory diagnostics. No official test file is opened, no λ is promoted as a newly qualified model, and unsuccessful runs remain in the denominator. The training log records the fraction of examples for which at least one normalized path maximum exceeds the penalty margin 0.9.

State fidelity is measured with the peak hidden-state RMSE defined in Section 6.12, comparing polynomial and exact activations of the same fine-tuned checkpoint. Each seed contributes a higher-order-statistic 95th percentile; the reported summary averages those five values. This measure differs from distillation error relative to the frozen teacher. The independent norm diagnostic uses the original fixed-weight checkpoints and the deterministic sample specified in Section 5.6; it does not use the fine-tuned weights. Protected original files are checked for unchanged hashes.

### 6.15. Additional Calibration and Dynamics Studies

Polynomial degrees (3, 3), (3, 5), (3, 7) and (5, 7) are evaluated with the five frozen TwoPatterns checkpoints on the original 99 selection and 104 qualification rows. We compare fixed domains (8, 6) with selection-only recalibration over the same 49-domain grid, retaining all seeds and keeping official tests closed. Four actual target-profile CKKS cell probes measure residual depth at hidden dimension 32 and batch one; these probes contain no refresh. Threshold replay independently varies the historical domain limit and shortlist tolerance, the prospective selector’s accuracy window, and the qualification utility tolerance, with α = 0.05 fixed. All are retrospective sensitivity studies with preserved original locks.

The candidate sensitivity study evaluates 49 pairs × five seeds × two original reliability tasks. It computes complete float64 GRU Jacobians on the first min(16, n) selection rows of each task. Full windows of lengths 8, 16 and 32 start every eight steps; each trajectory contributes its maximum log10 product norm, then the checkpoint p95 is used. This sampled-window extension is distinct from the original shortlist diagnostic. Jacobians are checked against automatic differentiation for all ten models. Within-seed Spearman correlations compare selection metrics with the previously recorded float32 qualification p95 peak state RMSE. Accuracy, domain, direct-error, Jacobian and domain-plus-Jacobian selectors share the two-point accuracy window. The comparison is retrospective and does not reopen official tests.

Four additional tasks are fixed before acquisition: ECG200 (100/100 TRAIN/TEST, length 96, two classes), ItalyPowerDemand (67/1029, 24, two), Plane (105/105, 144, seven) and Strawberry (613/370, 235, two). Five seeds use the common architecture and training budget, with class-stratified TRAIN fractions of 60%/15%/15%/remainder for training, early stopping, selection and qualification, rounding down within class (split seed 20260919). A relevance flag requires mean exact selection macro accuracy ≥75% and ≥20 points above chance, plus a worst-seed candidate-violation span ≥10 points with at least three distinct values. All four tasks are retained regardless of this flag. Original 49-pair selections are locked before qualification and the once-only official evaluation.

After the original-grid results, a separate exploratory grid uses Sigmoid bounds {1,2,3,4,5,6,8,10,12} and Tanh bounds {1,2,3,4,5,6,8}, identically on all four tasks. These 63 pairs reuse fixed weights and TRAIN-derived partitions and keep official tests closed. This wider range of candidate domains exposes coverage variation but does not constitute a second independent prospective study. Paired dynamics diagnostics independently use all original selection rows on Wafer and ElectricDevices, five seeds, and three fixed domain pairs per task. They replace only z, r or n with its exact activation while preserving weights and inputs, and record the first preactivation-domain excursion, hidden magnitudes above 2 and 10, and the first nonfinite state. Gate ties are retained. These are mechanistic counterfactuals, not deployable HE substitutions.

### 6.16. Additional Encrypted and System Experiments

The target-profile short run uses the frozen TwoPatterns seed-11 model and source 70, hidden dimension 32, batch one, and 32 active slots. It processes four steps, performs an actual refresh after each, and evaluates the trained eight-bin head using adaptive prefix pooling: for this four-step prefix, each state contributes to two bins, so all eight bins are defined. This differs from the full-length eight-bin partition in Section 4.2 and verifies a prefix computation rather than the classification outcome of a complete 128-step sequence. Residual conjugate-invariant log N = 15 and standard Bootstrapping log N = 16 use log_2_(QP) ≈ 638 and 1520, main secret H = 192, ephemeral secret H = 32, and scale 2^40^. One independent Bootstrapping operation warms each process. Verification-only intermediate decryption never feeds the encrypted recurrence.

The matched baseline is a standard trained LSTM with cubic i/f/o gates and degree-seven candidate and cell-output Tanh, updating c = f⊙c + i⊙g and h = o⊙Tanh(c). Each step refreshes c from level 5 to 10, then h from level 6 to 10. GRU and LSTM share preprocessing, splits, five training seeds, optimization budget, hidden dimension, batch, four-step input, eight-bin prefix head, CKKS moduli, secrets, BSGS kernels, key construction and encoding policy. Their parameter counts are 4772 and 6148. LSTM calibration uses the same 49-pair grid; the predeclared finite-accuracy fallback selects (6, 5) when no pair meets both domain screening and the accuracy window. It supplies an executable baseline rather than a domain-qualified deployment model.

Matched runs use an Intel Core i7-1165G7 with 16 GB random-access memory (RAM), Windows, Go 1.24.8 and Lattigo v6.1.1; GOMAXPROCS = 2, GOGC = 30 and GOMEMLIMIT = 6144 MiB. Three pairs run in GRU/LSTM, LSTM/GRU, GRU/LSTM order after other experiments finish. Circuit time includes matrix encoding and evaluation, nonlinear operations, actual refresh, and the encrypted head. Client encryption, verification decryption, file I/O, forced end-of-step garbage collection (GC) and setup/warm-up are excluded and reported through separate loop/process timings. Instrumented API counts include polynomial internals; bootstrap internals are excluded from residual counts and bootstrap calls are counted separately. Key sizes use BinarySize, and memory uses Windows process working set and private bytes.

For full-sequence numerical validation, twelve distinct seed-11 qualification inputs exclude historical sources 70 and 533 and cover four classes with three sequences each. Six are ordinary and six are relatively high-risk according to historical domain scores or state error. The fixed selection rule takes the minimum and maximum historical domain score within each class, then another low-score example in the first two classes and a high-state-error example in the other two, without replacement and with source-index tie-breaking. This purposive design probes a wider observed difficulty range; it is not a random draw from a deployment population. All selected historical domain scores are below one. The structural log N = 12 profile uses 64 replicas of each input for packing, counted as one sequence, and 128 cycles of 9 → 1 → 9. Bootstrap-induced error compares decoded values immediately before and after the same refresh. One standby-interrupted attempt is retained and rerun with the identical input and binary; only twelve completed sequences enter the plotted sequence distributions.

The timing investigation aligns original per-component wall-clock records with Windows Kernel-Power events and repeats the two affected full-sequence cases three times each. Each process includes one full-cell warm-up; GOMAXPROCS = 2, GOGC = 100 and the Go memory limit is off. Central processing unit (CPU), memory, GC and power events are monitored. Structural-profile timing repeats, and target-profile matched comparisons have separate resource policies and denominators.

## 7. Results

The main fixed-weight outcomes are reported in Table 7. Calibration results, prospective comparisons, retrospective diagnostics, and encrypted arithmetic checks retain the evidence boundaries defined in Section 6.13.

Values are means across five locked checkpoints unless a worst-seed result is identified. Peak state error $E_{i}$, defined in Section 6.12, is the maximum hidden-state RMSE over one trajectory relative to the same checkpoint with exact activations. The table reports the mean across checkpoints of its sample 95th percentile.

### 7.1. Frozen Qualification Outcomes

TwoPatterns selects sig8_tanh6 with a maximum selection violation rate of 3.030% and finite ordinary and doubled-length selection trajectories. Its 104 qualification rows per seed give rank scores from 0.814725 to 0.967613, all below one. These are empirical rank outcomes; task utility is assessed separately. Wafer and ElectricDevices retain their original abstentions and unopened official test files. The subsequent gate interventions in Section 7.15 explain the mechanisms behind these screening outcomes and identify candidate-state control as a useful direction.

### 7.2. Domain Coverage and Actual Trajectory Error

In the original two-task prospective comparison, all three selectors coincide on SyntheticControl, and accuracy and domain selection coincide on ChlorineConcentration. Direct-error selection reduces ChlorineConcentration test tail state error by 61.173%, with improvement in all five seeds; mean domain violation changes from zero to 0.0521%. All locked test candidates remain finite at ordinary and doubled length, and none has peak state RMSE above 0.5. These observations support direct fidelity assessment, while the weak exact ChlorineConcentration model and coincident domain choices delimit this original comparison. The additional tasks and informative domain-grid extension are reported separately in Section 7.14.

Selection-set direct error ranks qualification error consistently, with within-seed Spearman correlations from 0.9854 to 0.9990. Domain violation is constant on SyntheticControl and therefore has undefined correlation; the three estimable ChlorineConcentration values are −0.2969, −0.3670 and −0.3897. The original domain-proxy criterion is thus unmet, whereas direct replay supplies a stronger observed predictor. These are descriptive candidate-dependent associations. Section 7.13 adds the complete Jacobian comparison over the same candidate families.

The retrospective TwoPatterns comparison demonstrates the specific effect of domain screening: moving from sig6_tanh5.5 to sig8_tanh6 lowers mean violations from 79.231% to 0.577%. On the reused qualification rows, mean tail state RMSE changes from 0.573960 to 0.616972, the fraction above 0.5 changes from 16.923% to 30.385%, and macro accuracy changes from 97.936% to 95.454%; four checkpoints have greater tail error. Reporting these quantities together distinguishes the large coverage improvement from trajectory fidelity, without treating reused rows as an independent test.

Recurrent fidelity is measured directly rather than inferred from domain coverage. For every evaluated sequence, the polynomial and exact-activation models use identical weights and inputs; their hidden states are compared at each time step, and the largest state RMSE over that trajectory is summarized by the checkpoint p95 defined in Section 6.12. The same-weight SyntheticControl comparison yields mean test p95 peak state RMSE of 0.107476. On ChlorineConcentration, direct-error selection lowers this test statistic from 0.194796 to 0.075633, a 61.173% reduction, while all locked ordinary and doubled-length test trajectories remain finite. The TwoPatterns replay shows that domain screening can improve interval coverage without lowering direct state error on reused qualification rows. Together, these observations validate the proposed measurement of recurrent fidelity and identify where selection criteria improve it.

### 7.3. Untouched TwoPatterns Test Result

The locked polynomial model achieves 92.431 ± 3.461% macro accuracy on the official TwoPatterns test, compared with 98.114 ± 1.026% for exact activations. All polynomial trajectories remain finite. The paired accuracy difference is 5.683 ± 3.414 percentage points; one of five seeds lies within a two-point tolerance. These mean ± seed-SD results report the fixed-degree model’s task performance without asserting accuracy equivalence.

The mean joint test violation rate is 0.830 ± 1.060%, with seed values from 0.050% to 2.625%, all below the frozen 5% selection threshold. This gives a positive test-time coverage result alongside the separately measured task accuracy. The degree study in Section 7.11 further examines approximation capacity on TRAIN-derived data without changing this locked test result.

### 7.4. Joint Calibration Ablation

Joint calibration improves the selection operating point: sig8_tanh6 achieves 95.038% macro accuracy with 3.030% worst-seed violations and finite trajectories, versus 69.276%, 100% and 131 nonfinite trajectories for the sig4_tanh3 reference. Neither one-axis family supplies an eligible pair. The widest sig12_tanh8 pair removes observed violations but has 71.192% selection and 65.665% qualification accuracy, illustrating why utility is assessed alongside coverage. This post-lock ablation uses no official-test values.

In the same-grid replay, adding the domain threshold changes the accuracy-only choice sig6_tanh5.5 (96.434% selection accuracy, 94.949% worst-checkpoint violations) to sig8_tanh6. It reduces worst-checkpoint violations by 91.919 percentage points for a 1.396-point accuracy change. All 49 candidates are finite at ordinary and doubled length. Only one pair enters the historical accuracy shortlist, so this replay identifies the domain filter’s effect; the expanded study in Section 7.13 separately tests whether Jacobian ranking changes choices.

Together these comparisons identify which filter changes the recorded choice and which numerical property improves. Domain screening provides explicit interval control, while direct-error replay checks the resulting recurrent trajectory. The broader candidate and dataset studies in Section 7.13 and Section 7.14 extend this distinction and identify additional positive operating points without revising the original locks.

### 7.5. Repeated Encrypted Closure on a Locked Model

The 64-step closure completes 64 actual Bootstrapping operations and verifies the level cycle 9→1→9. Every state is finite. Maximum trajectory RMSE against the plaintext polynomial reference is $1.0127\times 10^{-7}$, and maximum absolute error is $2.2753\times 10^{-7}$. Total elapsed time is 102.437 s, including 38.081 s for affine matrices, 3.159 s for nonlinear computation, and 59.823 s for Bootstrapping. Peak sampled Go heap is 964.784. These structural-profile measurements cannot be transferred to a secure deployment.

All four predetermined 128-step cases complete recurrence, eight encrypted temporal bins, and the trained head. All 512 refreshes preserve the level cycle, every prediction matches its plaintext polynomial reference, and all values remain finite. Maximum hidden trajectory RMSE is $7.83\times 10^{-6}$, maximum hidden-coordinate error is $2.26\times 10^{-5}$, and maximum logit absolute error is $1.49\times 10^{-5}$. Three cases exceed the diagnostic $10^{-5}$ logit-error target, so the experiment establishes prediction agreement on four reused cases rather than that precision target or encrypted test accuracy.

The original full-sequence runs reach a peak sampled heap of 1270.225 MiB. The two wall-clock steps above 4000 s are now aligned with Windows standby records, and controlled warm repetitions characterize the active-run timing (Section 7.17). Historical values are retained without constructing corrected latency by subtracting sleep. These structural-profile timings and replicated inputs have their stated implementation scope; target-profile cost is measured separately in Section 7.10.

In the separate 64-step structural run, component totals correspond to approximately 37.2% affine computation, 3.1% nonlinear computation, and 58.4% Bootstrapping, with about 1.3% remaining overhead. This motivates measuring refresh cost explicitly. The subsequent matched target-profile comparison confirms lower total circuit time for the one-state GRU schedule in all three paired repetitions, while reporting memory and shared-key storage separately.

### 7.6. Target Residual Circuit

Five executions of the intended conjugate-invariant residual profile consume eight levels. Median residual latency is 13.260 s, and median RMSE is $2.87\times 10^{-7}$. This verifies residual depth and precision at logN=15 on synthetic hidden-dimension 64, batch-size 512 inputs. It is not an encrypted task evaluation.

### 7.7. Pinned Threshold Security Conformance

The intended residual and refresh profiles have logQP=638 and 1520, leaving margins of 130 and 30 bits relative to pinned Lattigo sparse-secret references. Uniform-ternary Category 128 limits provide margins of 243 and 256 bits. The latter comparison uses a different secret distribution. These results support conformance to pinned profiles labeled for 128-bit security but do not replace an attack-by-attack estimate for the exact parameters.

### 7.8. Secondary Fine-Tuning Results

The exploratory TwoPatterns pilot reduces 41 ordinary qualification violations to zero across five constrained runs, with both arms obtaining 100% accuracy on the same reused rows. It supplies no independent qualification or official test result. PhalangesOutlinesCorrect completes all seeds, but the penalty remains inactive, and the paired weights are identical. Its teacher, direct polynomial, and fine-tuned mean test accuracies are 54.458%, 55.594%, and 56.588%, with no observed range or finite failure.

On UWaveGestureLibraryX, seed 55 provides a positive paired operating point: the domain penalty reduces ordinary-test violations from 143 to 43 (69.93%), with macro accuracy changing from 70.644% to 70.363%. Both models remain finite at ordinary and doubled length. Table 8 adds the same-weight trajectory comparison: at 315 steps, the maximum observed peak state RMSE decreases from 5.128323 to 2.567673 (49.93%), accompanied by smaller median and p95 errors. The 630-step replay shows the same pattern. The improvement is greatest in the maximum observed error; the central and p95 changes are modest. The previously recorded logits RMSE relative to the frozen teacher is 0.290530 versus 0.291485 and is a separate metric.

The replay reproduces the historical accuracy, domain scores and finite-trajectory counts. Seed 44 has identical paired weights and identical new trajectories; its original ordinary and stress nonfinite counts are retained. Seeds 11, 22 and 33 retain their original first-epoch nonfinite training outcomes.

### 7.9. Penalty-Weight Sensitivity on Reused TwoPatterns Data

Accuracy is the mean ± sample standard deviation over five seeds. Violations and nonfinite counts are totals over five evaluations of the same 104 rows, not 520 independent population samples. State error compares polynomial and exact activations of the same fine-tuned checkpoint. Table 9 is exploratory and supplies no new independent qualification or official-test result.

**Figure 1:**
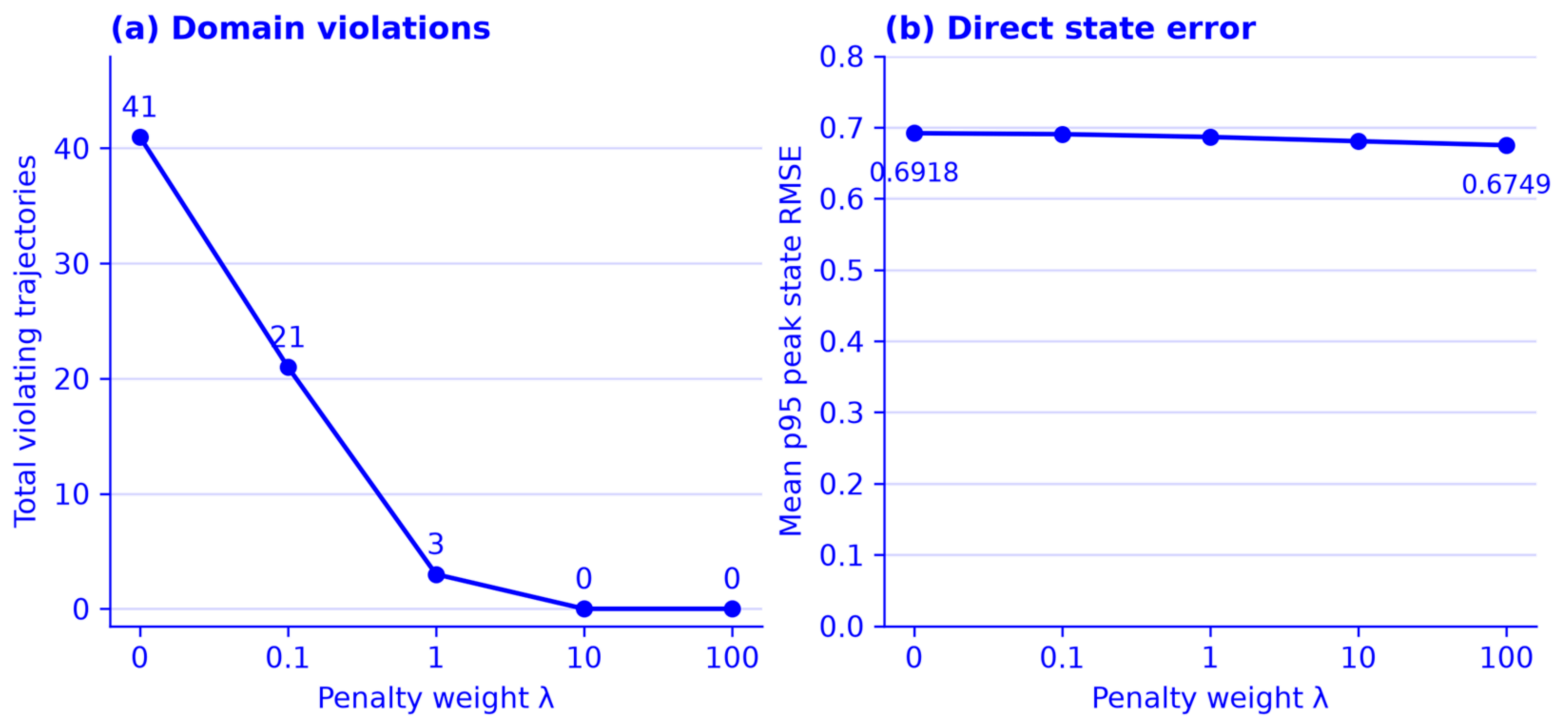
Penalty-weight sensitivity on the reused TwoPatterns qualification rows. (**a**) Total domain violations over the five checkpoints. (**b**) Mean checkpoint p95 peak state RMSE against exact activations of the same fine-tuned weights. λ values are displayed as ordered categories; the error axis begins at zero. All settings retain 100% qualification macro accuracy and finite ordinary and doubled-length trajectories. These are exploratory comparisons, not independent population estimates.

All 25 scheduled runs completed successfully. On the reused qualification rows, each λ retains 100% macro accuracy in every seed, and no ordinary or doubled-length trajectory is nonfinite. The observed domain-violation totals decrease from 41 at λ = 0 to 21, 3, 0, and 0 at λ = 0.1, 1, 10, and 100, respectively. The λ = 0 and λ = 10 aggregates reproduce the original pilot’s 41-to-zero contrast. This supports a graded response to the penalty and successful domain control at both tested larger weights. Figure 1 illustrates the reductions in domain violations and mean p95 state RMSE as λ increases.

Mean p95 state RMSE also decreases, from 0.691806 at λ = 0 to 0.680566 at λ = 10 and 0.674929 at λ = 100. The improvements are modest, approximately 1.6% and 2.4%, respectively, and are reported separately from the greater change in domain violations. The fraction of training examples with an active penalty in the final epoch falls from 35.65% at λ = 0 to 6.26% at λ = 10 and 2.31% at λ = 100, confirming that this task exercises the penalty mechanism.

The reused selection rows provide a consistent complementary diagnostic: mean macro accuracy remains 97.815%, and violation totals across the five checkpoints fall from 40 at λ = 0 to 24, 4, 4, and 1 over the increasing weights. Thus, zero observed qualification violations do not mean that every evaluated sequence satisfies the fitting domains. The results support the original λ = 10 setting within this exploratory comparison and show a similar qualification outcome at λ = 100; they do not establish a universal optimum or change the fixed-weight official-test result.

### 7.10. Target-Profile End-to-End Execution and Matched LSTM Cost

The intended profile audited against pinned 128-bit sparse-secret references completes four encrypted GRU steps, four recurrent refreshes, adaptive eight-bin prefix pooling, and the trained head. Every step follows 10 → 2 → 10. Maximum post-refresh hidden RMSE is 1.336 × 10^−6^; logit RMSE is 1.974 × 10^−6^ and maximum logit error is 2.910 × 10^−6^ relative to the same plaintext polynomial prefix circuit. Its prediction agrees with that arithmetic reference. Including one warm-up, five actual refreshes execute; whole-process time is 168.744 s and peak working set is 6.904 GiB. This directly validates short-prefix target-profile execution. Security evidence comes from the separate parameter audit in Section 7.7, and full-horizon target-profile task accuracy is not measured by this four-step check.

Table 10 gives the matched three-repeat system comparison. All six encrypted runs are finite, meet the predeclared 10^−4^ maximum absolute error tolerance and agree with their own plaintext polynomial prefix predictions. GRU is faster in every paired circuit measurement. The ratio of LSTM to GRU median four-step circuit times is 1.489, corresponding to 32.8% lower GRU latency. Peak memory and serialized evaluation-key storage are similar because both implementations reuse the same key set. The measured saving is in executed operations and refresh calls under this schedule.

The shared output head additionally uses 256 ciphertext–plaintext multiplications, 256 rescales and 80 rotations per complete four-step run. Bootstrap-internal operations are excluded from the residual counts in Table 10. Four-step component-time medians for GRU/LSTM are 79.609/83.803 s for affine computation, 6.644/14.083 s for nonlinear operations, 121.814/233.455 s for refresh and 17.082/11.542 s for the head. Component medians need not sum to the median total. The ranges retain observed runtime variation; three repetitions establish this measured operating point rather than a hardware-independent speed factor.

Task quality is assessed separately on full 128-step plaintext qualification trajectories. Across five seeds, exact GRU/LSTM macro accuracy is 99.64 ± 0.80%/99.45 ± 0.50%, and polynomial accuracy is 95.45 ± 4.41%/83.37 ± 6.94%; both are finite at ordinary and doubled length. The LSTM (6, 5) fallback has not passed the original domain eligibility rule. These results retain the baseline’s actual approximation behavior and distinguish it from the small CKKS implementation error. Four-step prefix agreement is an arithmetic check, not full-task classification accuracy or a PPLSTM reproduction.

### 7.11. Polynomial Degree and Depth Tradeoff

The frozen TwoPatterns degree comparison supports (3, 7) as an eight-level operating point with higher qualification macro accuracy than the shallower alternatives at fixed domains. Raising Tanh degree from five to seven changes mean qualification accuracy from 87.88% to 95.45% without increasing the measured eight-level residual depth; raising Sigmoid degree to five reaches 97.57% but uses nine levels. Recalibration improves some lower-degree alternatives, showing that both polynomial capacity and fitted domain matter. These are reused qualification rows, and the degree probes measure one target-profile cell rather than repeated refresh for every degree variant. The original locked official-test result is unchanged. As shown in Table 11.

The four target-profile cell probes consume levels 10 → 3, 10 → 2, 10 → 2, and 10 → 1, respectively; each has maximum coordinate error below 10^−4^ against its own plaintext polynomial cell. Relative to the recorded minimum refresh-input level of one, their residual margins are two, one, one, and zero levels. These measurements show how degree choice changes headroom within a fixed parameter chain. Repeated refresh and complete output evaluation are directly measured for (3, 7); they have not been established for the other degrees or for enlarged modulus chains. This distinction explains why the eight-level (3, 7) choice is a tested operating point rather than a universal depth optimum.

### 7.12. Threshold Sensitivity and Empirical Qualification

The thresholds are declared engineering budgets: 5% limits observed selection-domain excursions; the historical 0.5-point shortlist tolerance compares with the best domain-eligible candidate; the prospective two-point selection window compares with the best finite candidate; and the five-point utility tolerance compares with the same checkpoint’s exact model. They have different reference quantities. The rank parameter α = 0.05 is separate from the selection-domain limit. Table 12 summarizes the retrospective replay, which provides local sensitivity evidence for these original choices.

The local invariance of the TwoPatterns choice and the 90/90 prospective replay records supports a reproducible operating region. At tighter historical domain limits or larger shortlists, missing original Jacobian records prevent a complete replay of the final historical ranking; these entries are retained as unresolved rather than assigned an imputed choice. Wafer and ElectricDevices retain their original abstentions across the tested limits. Changing the utility tolerance changes the pass decision for some fixed ChlorineConcentration checkpoints, not their performance. The reported pass counts remain sample assessments, with no population-confidence interpretation.

### 7.13. What Candidate Jacobians Predict

The expanded 490 candidate–seed study gives the sensitivity metrics a direct empirical test beyond a one-candidate shortlist. Table 13 shows that selection-set direct state error has Spearman correlation above 0.98 with qualification error in all ten dataset–seed groups. This strong cross-partition ranking consistency provides practical evidence for direct fidelity measurement. Product norms have negative associations with observed approximation error in these candidate families, clarifying that a smaller propagation factor alone does not imply a smaller injected approximation defect.

Under the same accuracy window, the Jacobian and domain-plus-Jacobian selectors choose (11, 7.5) on SyntheticControl, so the diagnostic now changes the candidate. Its qualification macro accuracy and p95 state RMSE are 88.00% and 0.2482, versus 91.54% and 0.1088 for the (6, 5) accuracy/domain/direct-error choice. On ChlorineConcentration, Jacobian and accuracy selectors coincide at (12, 8); direct-error selection at (6, 5) reduces mean qualification p95 RMSE from 0.1942 to 0.07544 while increasing macro accuracy from 30.55% to 32.29%. This supports complementary roles: Jacobians explain propagation, whereas direct replay measures the combined propagation and local-defect outcome. These shared-data associations are descriptive, not independent-sample significance tests.

### 7.14. Additional Datasets and Informative Domain Variation

Table 14 broadens the official-test evidence with all four predeclared tasks. The original-grid polynomial model attains 76.04 ± 1.83% macro accuracy on ECG200 compared with 74.69 ± 1.93% for exact activations; Plane reaches 79.17 ± 6.26% under accuracy/domain selection and 79.63 ± 5.42% under direct-error selection. The five-seed results and retained Strawberry abstention make the scope of each selector visible.

Exact selection macro accuracies are 94.00%, 77.50%, 78.57% and 72.88% for ECG200, ItalyPowerDemand, Plane and Strawberry, respectively. The first three meet the model-quality flag but have constant zero domain violation over the original broad grid; Strawberry has variable coverage but falls below the quality threshold. Consequently, none meet both relevance conditions in the initial grid. The separate uniform 63-pair exploration yields violation spans of 100 percentage points and 7, 4, 3 and 8 distinct values, respectively, so the first three now combine reasonable exact models with informative coverage variation. This motivates the clearly labeled exploratory comparison in Table 15.

On ECG200 and ItalyPowerDemand, domain screening lowers mean qualification p95 state error by 74.1% and 94.6% relative to accuracy selection, with accuracy gains of 1.25 and 2.50 points. These positive results identify tasks and domain families in which coverage helps selection. Plane retains essentially the same error under domain selection, while direct error produces a clearer improvement; Strawberry retains its domain abstention. Coverage and direct error are therefore complementary diagnostics. The extension was designed after the initial results and reuses qualification rows; its gains are exploratory rather than independent test improvements.

### 7.15. Gate-Level Mechanisms of Recurrent Growth

**Figure 2:**
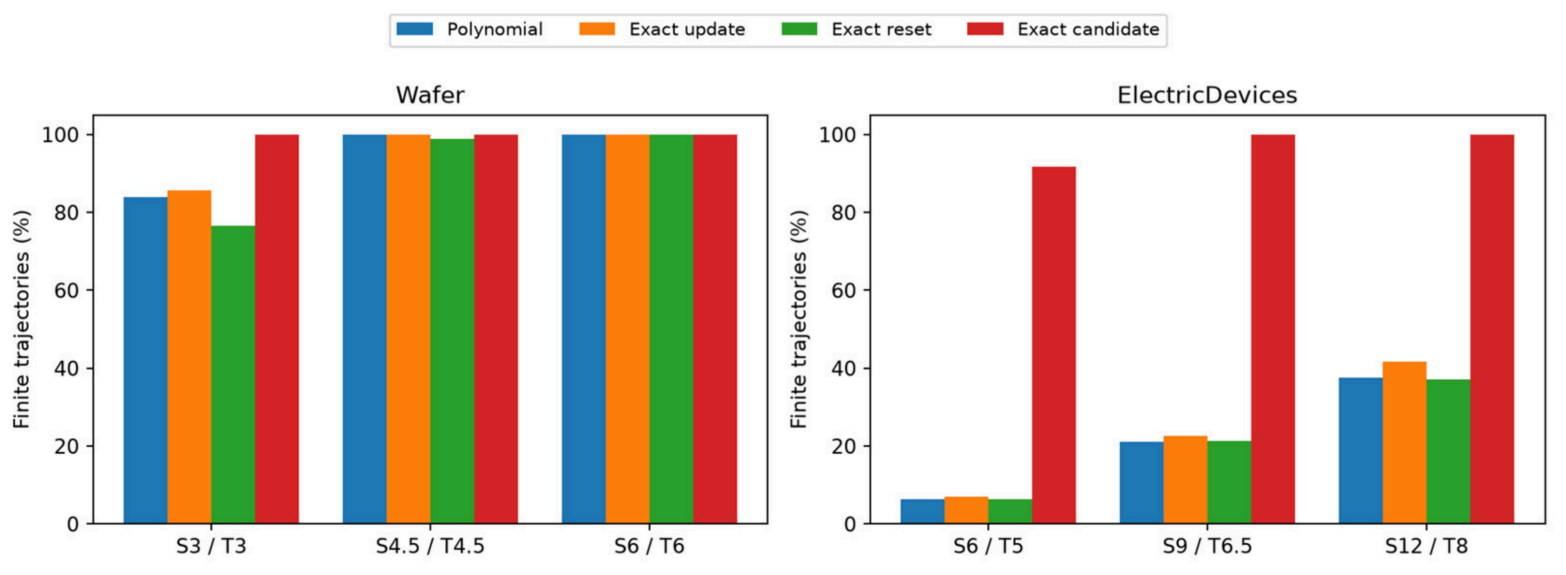
Paired gate interventions on the original selection partitions, five frozen seeds and three domains per task. Finite percentages count 495 Wafer and 4445 ElectricDevices sequence–seed instances per setting, with repeated inputs across seeds. Exact single-gate activations are diagnostic counterfactuals; all other operations remain polynomial.

The paired interventions identify the candidate-state activation as the most effective tested control point. Figure 2 reports the finite-trajectory fraction for all four variants. Replacing only n with exact Tanh gives finite trajectories for all three Wafer settings and for the middle and widest ElectricDevices settings. At the narrowest ElectricDevices setting, nonfinite instances decrease from 4160 to 367 out of 4445 sequence–seed evaluations. Exact z or r alone provides substantially less consistent improvement. These controlled changes explain the model dynamics without changing weights or selecting a new encrypted deployment.

Table 16 shows when preactivation excursions and hidden-state growth occur. At the narrow Wafer setting, first excursions most often involve all three gates together (264/495), followed by z alone (107/495). At the widest ElectricDevices setting, n alone is first in 2664/4445 instances and z + n are simultaneous in 877. The first crossing need not identify the most useful intervention, because gates are recurrently coupled.

The recurrence explains the useful intervention: if z stays in [0, 1] and n in [−1, 1], the hidden update is a coordinatewise convex combination, so h_0_ = 0 preserves |h|∞ ≤ 1. The exact candidate Tanh directly controls one of these factors. The exact update alone cannot bound an extrapolating polynomial candidate, while the exact candidate alone still leaves the polynomial update gate unconstrained. The remaining narrow-domain ElectricDevices cases therefore motivate joint range control. Recorded recurrent-matrix spectral norms describe amplification potential, but no matrix intervention establishes them as a sole cause. The two broader Wafer settings are already finite; their historical rejection concerns domain coverage rather than numerical divergence.

### 7.16. Distributional Validation on Twelve Additional Full Sequences

All twelve selected 128-step structural-profile sequences complete recurrence, pooling, and the trained encrypted head. The completed runs execute 1536 recurrent refreshes plus twelve separate warm-ups, preserve the 9 → 1 → 9 level cycle, satisfy the predeclared 10^−3^ maximum coordinate-error gate, and retain finite states. The encrypted output prediction agrees with the same-weight plaintext polynomial reference in 12/12 cases. This verifies arithmetic fidelity for these selected sequences; it does not establish agreement with the true class or target-security execution over 128 steps.

**Figure 3:**
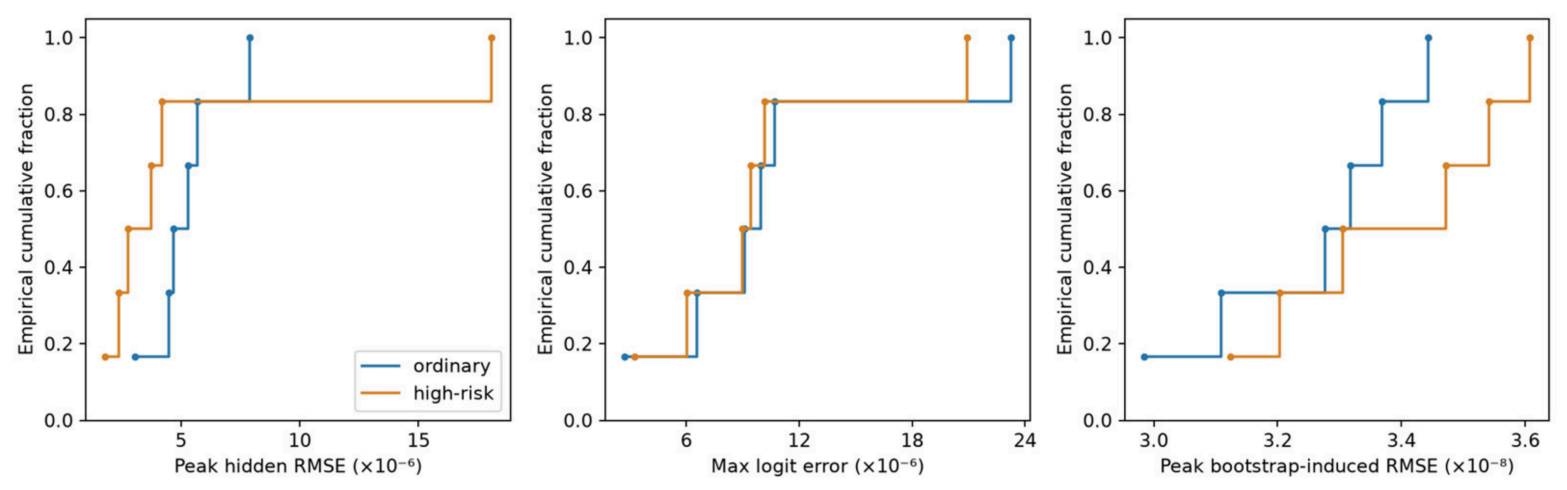
Empirical cumulative distributions over twelve distinct completed 128-step structural-profile sequences: per-sequence peak hidden RMSE, maximum logit absolute error, and peak bootstrap-induced RMSE. Each selected stratum contains six sequences. The 64 identical packed replicas and 128 dependent time steps per sequence do not increase the sequence sample size. Errors compare encrypted execution with the same plaintext polynomial circuit; the curves describe the selected inputs, not a randomly sampled input population.

Figure 3 reports the empirical cumulative distribution of one error value per completed sequence. For a metric E and n = 12 distinct inputs, the plotted value at e is the fraction of sequences with E ≤ e; ordinary and relatively high-risk strata each have n = 6. Across all twelve, median/maximum peak hidden RMSE is 4.340 × 10^−6^/1.805 × 10^−5^, and median/maximum logit absolute error is 9.257 × 10^−6^/2.323 × 10^−5^. Peak bootstrap-induced RMSE per sequence has a median 3.311 × 10^−8^ and a maximum 3.608 × 10^−8^. The separate pooled summary over 1536 dependent steps has a bootstrap-induced RMSE median 2.469 × 10^−8^ and a p95 3.041 × 10^−8^; it is a within-run arithmetic summary, not an independent-sequence sample.

The plotted sequence values and reported quantiles should be read with their small denominators: higher-order-statistic p90 and p95 within a six-sequence stratum commonly equal the maximum. The risk label reflects historical domain score or state error and does not assert an actual out-of-domain input; scores range from 0.70 to 0.97 across the selected examples. The interrupted attempt and standalone warm-ups do not enter the twelve completed-sequence curves. These full-horizon descriptive results use the intentionally insecure structural profile; target-profile evidence remains the separate four-step execution in Section 7.10. The twelve rows in the archived sequence_metrics.csv have unique source indices and reproduce the plotted per-sequence medians and maxima for hidden RMSE, logit error, and bootstrap-induced RMSE; the same rows record 12/12 encrypted-versus-polynomial prediction agreement. This arithmetic check uses the locked structural-profile cases and adds no new input sample.

### 7.17. Explained Timing Outliers and Controlled Warm Repeats

The two historical >4000 s components align with Windows Kernel-Power 506/507 standby sessions triggered by the lid. The 4129.845 s affine component (seed 11, source 533, step 96) overlaps 4127.325 s of recorded sleep; the 4237.595 s refresh component (seed 55, source 70, step 113) overlaps 4234.470 s. Standby accounts for approximately 99.94% and 99.93% of those wall-clock intervals. This identifies a host power-state cause for the anomalous records. The raw timings are preserved; subtracting sleep is not used to manufacture clean benchmark samples.

Six newly warmed full-sequence runs (three per affected case) execute 768 recurrent steps plus six cell warm-ups, with finite states, the expected level cycle, and 6/6 plaintext-polynomial prediction agreement. Table 17 summarizes complete-iteration timing, which includes loop verification overhead and differs from the original three-component sum. No standby event occurs during these controlled repeats. Sampled free memory remains at least 5.670 GiB; peak working set is 1.215–1.240 GiB. The 768 forced-GC calls have 87.15 ms total recorded stop-the-world pause and 17.594 ms maximum, providing a separate resource check.

The repeat evidence establishes an active-run timing distribution with a maximum full iteration below 2.61 s for these cases, while the power-event audit explains the historical stalls. The target-profile matched comparison in Table 10 uses its own identical resource policy and remains the basis for the GRU–LSTM system-cost claim.

### 7.18. Step-Count Sensitivity on Frozen Inputs

Table 18A,B compare recurrent horizons with the checkpoint, coefficients, and twelve selected qualification inputs held fixed. Table 18A summarizes dependent prefixes of archived structural-profile encrypted runs; Table 18B compares the frozen polynomial and exact-activation models in plaintext, including a 256-step repeated-input replay.

All twelve structural-profile encrypted trajectories remained finite and preserved the measured 9 → 1 → 9 ciphertext-level cycle at every evaluated horizon. Median per-sequence peak hidden-state RMSE against the plaintext polynomial circuit was 1.04 × 10^−7^ by step 32, 1.08 × 10^−6^ by step 64, 3.51 × 10^−6^ by step 96, and 4.34 × 10^−6^ by step 128. The largest observed coordinate error by step 128 was 6.99 × 10^−5^, below the predeclared 10^−3^ structural-profile gate. These within-prefix peaks summarize nested horizons rather than independent runs or a per-step growth rate.

In the separate plaintext comparison, all twelve trajectories remained finite and within the frozen fitting domains through the 256-step replay. Median per-sequence peak polynomial-versus-exact hidden-state RMSE was 0.300 at 64 steps and 0.454 at both 128 and 256 steps. One selected input had a second-pass peak of 0.437, up from 0.296 at 128 steps; relative increases for the other eleven were at most 0.11%. Thus, repeated execution preserved finiteness and domain coverage on these inputs, while state error could depend on the horizon. These selected qualification inputs and the repeated-input stress case do not measure natural-length task accuracy.

## 8. Conclusions

OneBoot GRU combines locked, joint polynomial-domain calibration with an executable CKKS recurrent circuit. Its degree-three Sigmoid and degree-seven Tanh fit an eight-level cell followed by one hidden-state refresh. A profile audited against pinned 128-bit sparse-secret references completes four-step recurrent execution, adaptive prefix pooling, and the trained head. In three matched four-step comparisons, the implemented GRU has median circuit latency 226.343 s versus 336.981 s for the polynomial LSTM, a 32.8% reduction under the stated hardware and shared kernels. All six encrypted runs remain finite and agree with their own plaintext polynomial prefix predictions.

Under the separate structural profile, twelve distinct 128-step sequences complete 1536 recurrent refreshes and encrypted readouts with 12/12 prediction agreement relative to the plaintext polynomial circuit. Maximum hidden-state RMSE and logit absolute error are 1.81 × 10^−5^ and 2.32 × 10^−5^. The empirical cumulative distributions summarize these selected cases, with each distinct input counted once. Controlled warm repeats and host power-event records explain the earlier timing outliers without changing the raw measurements. These results extend long-horizon arithmetic evidence while keeping its security scope distinct from the target-profile short run. The fixed-input step-count analysis also confirms finite structural-profile encrypted prefixes at 32–128 steps and finite, in-domain plaintext replay through 256 steps on the twelve selected cases.

The model comparisons show why domain coverage and direct state fidelity should be assessed together. TwoPatterns attains finite official-test trajectories with 0.830% mean domain violations and 92.431% polynomial macro accuracy, compared with 98.114% for its exact-activation reference. SyntheticControl attains 89.067% versus 90.133%. On reused TwoPatterns qualification rows, a large coverage gain does not improve tail state error; a separately labeled exploratory grid finds 74.1% and 94.6% lower qualification tail error on ECG200 and ItalyPowerDemand. Complete Jacobians, gate interventions, and paired exact-versus-polynomial trajectories help interpret these different outcomes without treating sampled coverage as a stability certificate.

The measured results support the stated short target-profile execution, long structural-profile arithmetic checks, and dataset-specific calibration outcomes. The security audit checks conformance to pinned sparse-secret references and does not include a fresh attack-by-attack estimate for the exact tuple. The 128-step sequence curves are descriptive because inputs were selected by a fixed difficulty rule; qualification decisions and exploratory gains are empirical sample assessments. The matched cost advantage applies to the two implemented recurrent graphs and their declared resource policy. These distinctions keep the positive operating points reproducible and interpretable.

The framework provides an auditable route from coupled domain selection to encrypted recurrent execution. Direct state-error measurement, locked model identities, verified derivatives, and complete encrypted traces make its numerical and system behavior inspectable. Longer target-profile task runs, an independent sample for exploratory domain improvements, and bounded candidate-state designs are useful next steps. The present evidence establishes working configurations and clearly identifies the remaining validation needed for broader deployment claims.

## Figures and Tables

**Table 1:** Related approaches and the specific comparison supported here.

Approach	Model/Arithmetic	Adaptation or Continuation	Relation to This Study
CryptoRNN [7]; Podschwadt and Takabi [8]	Encrypted recurrent prediction	Approximated nonlinearities	Prior noninteractive recurrent inference
CryptoGRU [9]	GRU; HE plus garbled circuits	Hybrid nonlinear evaluation	Different interaction model
PPLSTM [6]	LSTM; CKKS	State refresh and RMSNorm	Architectural context; matched standard-LSTM experiment in Section 7.10
AESPA [17]	Private neural inference	Hermite activation and normalization	Prior low-degree adaptation
AutoFHE [18]	CNN; RNS-CKKS	Mixed degrees and refresh placement	Prior accuracy–cost co-optimization
OneBoot GRU	GRU; CKKS	Fixed degrees; joint domain screening; one state refresh	Separates coverage, state fidelity and task utility

**Table 2:** Separate meanings of reliability and their evidence requirements.

Property	Quantity or Condition	Evidence and Limitation
Input confidentiality	Threat model and exact HE parameters	Target-profile audit; insecure structural runs do not establish security
Repeated execution	Level and scale restored after each cell	Measured level cycles; does not bound state error
HE arithmetic fidelity	Decoded CKKS versus plaintext polynomial outputs	Hidden-state and logit errors for specified encrypted cases
Recurrent fidelity	Polynomial versus exact-activation states with the same weights	Direct peak state RMSE and Jacobian diagnostics; coverage is not a substitute
Task utility	Accuracy loss relative to the declared reference	Separate tolerance and failure accounting; weak references remain a limitation

**Table 3:** Sampled state-dependent bound verification on the original TwoPatterns models.

Seed	p95 ‖JF‖_2_	p95 Bound	Median Bound/Norm	Violations
11	1.197	4.487	2.507	0
22	1.221	4.401	2.547	0
33	1.217	4.578	2.383	0
44	1.208	4.001	2.383	0
55	1.243	4.225	2.454	0

**Table 4:** Datasets, Experimental Roles, and Official Test Access.

Dataset	Role	Candidate Design or Intervention	Official Test Access
Wafer	Calibration	Smaller 49-pair grid	Terminated when no candidate was eligible
TwoPatterns	Calibration, retrospective diagnostic, and exploratory fine-tuning pilot	Broader 49-pair grid and fixed sig8_tanh6 pilot	Once after the calibration gate only. No new test access for retrospective or pilot analyses
ElectricDevices	Calibration	Broader 49-pair grid	Terminated when no candidate was eligible
SwedishLeaf and FordB	Development	Model and diagnostic development	No independent confirmation claimed
SyntheticControl	Prospective reliability comparison	Broader 49-pair grid with three selectors	All locked nonabstaining selections under the research policy
ChlorineConcentration	Prospective reliability comparison	Broader 49-pair grid with three selectors	All locked nonabstaining selections under the research policy
PhalangesOutlinesCorrect and UWaveGestureLibraryX	Prospective fine-tuning transfer	Ordinary and domain-constrained arms	All locked variants under the research policy
ECG200, ItalyPowerDemand, Plane and Strawberry	Additional five-seed comparison	Original 49-pair grid; separate exploratory 63-pair extension	Original locked selections tested once; extension uses TRAIN-derived rows only

**Table 5:** Cryptographic Parameter Profiles and Evidence Scope.

Profile	Ring and Slots	Modulus and Secret	Bootstrapping Status	Evidence Supplied
Structural closure	Standard, log N = 12, 2048 complex slots populated with real values	Maximum level 9	Repeatedly measured	Intentionally insecure arithmetic and implementation check
Intended residual	Conjugate invariant, log N = 15, 32,768 real slots	log Q = 455, log P = 183, log QP = 638, sparse ternary Hamming weight 192	Four-step recurrence and actual target refresh measured	Eight-level residual plus trained encrypted prefix head; matched baseline
Intended refresh	Standard, log N = 16, 32,768 complex slots	log QP = 1520; main sparse secret H = 192; ephemeral H = 32	Complete keys allocated; actual refresh and warm repeats measured	Pinned 128-bit-target reference conformance; measured short-run latency and memory

**Table 6:** Qualification and test-access rules differ across the recorded studies.

Study	Selection/Qualification	Official Test Policy
Historical calibration	Selection: finite ordinary and doubled trajectories, ≤5% domain violations; qualification: empirical rank gate	Rejection terminates testing; accepted locked model tested once
Prospective reliability comparison	Frozen selectors; separate rank, ordinary-finiteness, stress-finiteness and utility checks	All locked nonabstaining selections evaluated, including qualification failures
Prospective fine-tuning transfer	Matched adaptation; models and failures retained before qualification	All locked variants evaluated under the research policy
Revision sensitivity and norm checks	Fixed λ grid and diagnostic sample; reused training-derived partitions	No official test access; no new qualification or deployment approval
Additional datasets	Initial 49-pair selections locked before qualification; same three selectors	Locked original selections tested once; exploratory 63-pair extension has no test access

**Table 7:** Main Fixed-Weight Calibration and Prospective Reliability Results.

Study and Dataset	Locked Selection	Exact Macro Accuracy	Polynomial Macro Accuracy	Observed Domain Result	Mean p95 Peak State Error	Qualification or Test Status
Calibration, Wafer	No eligible candidate	Not tested	Not tested	Lowest worst-seed selection violation 48.485%	Not applicable	Rejected before official test access
Calibration, TwoPatterns	sig8_tanh6	98.114%	92.431%	Mean test violation 0.830%	Not measured on the official test	Empirical rank passes under the implemented calibration rule. All test trajectories finite
Calibration, ElectricDevices	No eligible candidate	Not tested	Not tested	Worst-seed violation 100% for every candidate	Not applicable	Rejected before official test access
Prospective, SyntheticControl, all selectors	sig6_tanh5	90.133%	89.067%	Test violation 0%	0.107476	Complete qualification passed for 5 of 5 checkpoints
Prospective, ChlorineConcentration, accuracy and domain selectors	sig12_tanh8	34.365%	33.404%	Test violation 0%	0.194796	Complete qualification passed for 3 of 5 checkpoints
Prospective, ChlorineConcentration, direct error selector	sig6_tanh5	34.365%	34.174%	Mean test violation 0.0521%	0.075633	Complete qualification passed for 4 of 5 checkpoints

**Table 8:** UWaveGestureLibraryX seed-55 paired replay on 3582 previously evaluated test sequences. Each state-error statistic summarizes per-sequence peak hidden-state RMSE against exact activations of the same fine-tuned weights. The doubled horizon reuses the same sequences.

Steps	Metric	Ordinary	Penalty	Reduction
315	Median peak state RMSE	0.825409	0.819036	0.77%
315	p95 peak state RMSE	1.239074	1.236123	0.24%
315	Maximum peak state RMSE	5.128323	2.567673	49.93%
630	Median peak state RMSE	1.067155	1.065930	0.11%
630	p95 peak state RMSE	1.240519	1.237385	0.25%
630	Maximum peak state RMSE	5.128325	2.567675	49.93%

**Table 9:** Revision-stage λ sensitivity on the reused TwoPatterns qualification rows.

λ	Runs	Macro Accuracy (%)	Violations/520	Mean p95 State RMSE	Stress Nonfinite
0	5/5	100.000 ± 0.000	41	0.691806	0
0.1	5/5	100.000 ± 0.000	21	0.690438	0
1	5/5	100.000 ± 0.000	3	0.686468	0
10	5/5	100.000 ± 0.000	0	0.680566	0
100	5/5	100.000 ± 0.000	0	0.674929	0

**Table 10:** Matched target-profile GRU and polynomial LSTM: per-step residual counts and four-step system measurements. Time and memory are medians [minimum, maximum] across three runs.

Metric	OneBoot GRU	Polynomial LSTM
Refresh calls/step	1	2
Ciphertext × ciphertext/step	11	19
Ciphertext × plaintext/step	192	256
Ciphertext × scalar/step	9	16
Relinearizations/step	11	19
Rescales/step	206	280
Rotations/step	60	80
Four-step circuit (s)	226.343 [131.955, 232.428]	336.981 [295.670, 371.994]
Measured loop (s)	227.218 [132.410, 233.169]	337.894 [296.411, 372.958]
Whole process (s)	285.377 [172.256, 305.196]	383.652 [339.571, 447.804]
Setup and warm-up (s)	51.001 [38.972, 76.757]	44.887 [42.273, 73.846]
Peak working set (GiB)	6.919 [6.899, 6.925]	6.929 [6.912, 6.937]
Peak private bytes (GiB)	7.015 [7.007, 7.015]	7.018 [7.018, 7.018]
Residual/refresh keys (MiB)	308.012/5132.087	308.012/5132.087
Total server evaluation keys (MiB)	5440.099	5440.099
Public/client secret key (MiB)	7.0003/3.5001	7.0003/3.5001

**Table 11:** Degree ablation on five frozen TwoPatterns models. Accuracy is qualification macro accuracy (%, mean ± seed SD). Depth is measured with an actual target-profile CKKS cell probe.

Sigmoid/Tanh Degree	Fixed (8, 6)	Recalibrated Grid	Levels Consumed
3/3	58.02 ± 6.06	64.40 ± 7.91	7
3/5	87.88 ± 10.68	92.40 ± 8.52	8
3/7	95.45 ± 4.41	95.45 ± 4.41	8
5/7	97.57 ± 2.35	97.57 ± 2.35	9

**Table 12:** Replay of selection and qualification thresholds with original models and records fixed. pp denotes percentage points.

Check	Settings Replayed	Observed Result
TwoPatterns local selection	Domain 5, 7.5, 10%; shortlist 0.25, 0.5, 0.75 pp	Same (8, 6) in all nine combinations
Two prospective tasks, three selectors	Domain 1, 2.5, 5, 7.5, 10%; accuracy window 1, 2, 3 pp	Original candidates in 90/90 selector records
SyntheticControl qualification	Utility tolerance 3, 5, 7 pp	5/5 checkpoints pass for every selector
ChlorineConcentration qualification	Utility tolerance 3/5/7 pp	Accuracy/domain: 3/3/5 passes; direct error: 3/4/4 passes, each out of five

**Table 13:** Within-seed Spearman correlations with qualification p95 peak state RMSE across 49 candidates. Entries are mean ± seed SD; parentheses give the number of estimable seeds.

Selection Predictor	SyntheticControl	ChlorineConcentration
Jacobian product statistic	−0.779 ± 0.199 (5/5)	−0.781 ± 0.071 (5/5)
Sampled single-step norm	−0.718 ± 0.214 (5/5)	−0.781 ± 0.071 (5/5)
Domain violation rate	Undefined: constant (0/5)	−0.351 ± 0.048 (3/5)
Direct p95 state error	0.994 ± 0.005 (5/5)	0.997 ± 0.003 (5/5)

**Table 14:** Once-only official-test macro accuracy (%) for the four additional tasks, mean ± sample SD across five seeds. A/D/E denote accuracy/domain/direct-error selectors; shared candidates are evaluated once.

Task	Exact Model	Polynomial Model	Selector; Domain Pair
ECG200	74.69 ± 1.93	76.04 ± 1.83	A/D/E; (6, 5)
ItalyPowerDemand	78.69 ± 11.76	76.27 ± 11.81	A/D/E; (8, 5)
Plane	78.67 ± 5.00	79.17 ± 6.26	A/D; (6, 6.5)
Plane	78.67 ± 5.00	79.63 ± 5.42	E; (6, 5)
Strawberry	73.07 ± 9.72	63.98 ± 10.38	A; (6, 5)
Strawberry	73.07 ± 9.72	62.22 ± 8.23	E; (12, 5)
Strawberry	73.07 ± 9.72	Abstain	D; no eligible pair

**Table 15:** Exploratory 63-pair extension on reused qualification rows. Accuracy and checkpoint p95 peak state RMSE are mean ± seed SD. These are separate from the official test results in Table 14.

Task/Selector	Pair	Macro Accuracy (%)	p95 State RMSE
ECG200/accuracy	8, 3	69.75 ± 5.48	0.275 ± 0.409
ECG200/domain	5, 5	71.00 ± 4.37	0.071 ± 0.005
ECG200/direct error	3, 4	71.00 ± 4.37	0.047 ± 0.010
ItalyPowerDemand/accuracy	6, 2	71.67 ± 21.53	0.901 ± 1.077
ItalyPowerDemand/domain	6, 4	74.17 ± 18.96	0.049 ± 0.005
ItalyPowerDemand/direct error	6, 3	71.67 ± 21.53	0.077 ± 0.080
Plane/accuracy	1, 8	79.29 ± 11.06	0.178 ± 0.006
Plane/domain	4, 8	79.29 ± 11.06	0.178 ± 0.007
Plane/direct error	2, 4	81.43 ± 10.16	0.038 ± 0.002
Strawberry/accuracy	3, 3	69.08 ± 13.31	0.975 ± 1.373
Strawberry/direct error	2, 3	69.08 ± 13.31	0.584 ± 0.794
Strawberry/domain	—	Abstain	—

**Table 16:** Median first-event step on original polynomial trajectories, conditional on each event occurring. Denominators differ between columns; differences in column medians are not paired delays. “—” means the event is absent.

Task; Domains	First Domain Excursion	First |h|∞ > 2	First Nonfinite
Wafer; (3, 3)	21	23	25
Wafer; (4.5, 4.5)	22	23	—
Wafer; (6, 6)	34	—	—
ElectricDevices; (6, 5)	32	35	40
ElectricDevices; (9, 6.5)	36	41	50
ElectricDevices; (12, 8)	42	50	54

**Table 17:** Controlled timing under the structural profile: six warmed 128-step runs. Full-iteration quantiles pool 768 dependent steps and are descriptive.

Measurement	Observed Time (s)
Full iteration: median/p95/maximum	1.658/1.909/2.609
Step 96 affine: three repeats	0.571/0.569/0.606
Step 113 refresh: three repeats	1.553/1.000/0.959

**Table 18:** (**A**). Encrypted step-count sensitivity on twelve selected TwoPatterns qualification sequences. Archived structural-profile prefixes (log N = 12) are compared with the same plaintext polynomial circuit; each value summarizes twelve distinct inputs. (**B**) Plaintext step-count sensitivity on the same twelve selected inputs with identical weights. Polynomial states are compared with exact-activation states. At 256 steps, each original 128-step input is repeated once without resetting the state.

(**A**)					
Steps T	Finite/12	Cycle/12	Median Peak Hidden RMSE	Maximum Peak Hidden RMSE	Largest Peak Coordinate Error
32	12	12	1.04 × 10^−7^	2.76 × 10^−6^	9.34 × 10^−6^
64	12	12	1.08 × 10^−6^	6.24 × 10^−6^	1.84 × 10^−5^
96	12	12	3.51 × 10^−6^	1.81 × 10^−5^	6.99 × 10^−5^
128	12	12	4.34 × 10^−6^	1.81 × 10^−5^	6.99 × 10^−5^
(**B**)					
Steps T	Input	Finite/12	Excursions/12	Median Peak Hidden RMSE	Maximum Peak Hidden RMSE
64	Natural prefix	12	0	0.300	0.507
128	Natural full sequence	12	0	0.454	1.302
256	Full sequence repeated once	12	0	0.454	1.302

## Data Availability

Data are contained within the article.

## Abbreviations

AESPA	Accuracy Preserving Low-degree Polynomial Activation
BSGS	Baby-step giant-step
CKKS	Cheon–Kim–Kim–Song
CNN	Convolutional neural network
CPU	Central processing unit
FHE	Fully homomorphic encryption
GC	Garbage collection
GRU	Gated recurrent unit
HE	Homomorphic encryption
LSTM	Long short-term memory
PCMM	Plaintext–ciphertext matrix multiplication
PPLSTM	Privacy-preserving long short-term memory protocol
RAM	Random-access memory
RLWE	Ring learning with errors
RMSE	Root mean square error
RMSNorm	Root mean square layer normalization
RNN	Recurrent neural network
RNS	Residue number system
SD	Standard deviation
SHA-256	Secure Hash Algorithm, 256-bit
SIMD	Single instruction, multiple data
TFHE	Fast Fully Homomorphic Encryption over the Torus
UCR	University of California, Riverside

## References

[1] Cheon J.H., Kim A., Kim M., Song Y. (2017). Homomorphic Encryption for Arithmetic of Approximate Numbers. Advances in Cryptology—ASIACRYPT 2017.

[2] Cho K., van Merriënboer B., Gulcehre C., Bahdanau D., Bougares F., Schwenk H., Bengio Y. (2014). Learning Phrase Representations Using RNN Encoder–Decoder for Statistical Machine Translation. Proceedings of the 2014 Conference on Empirical Methods in Natural Language Processing.

[3] Lei J., G’Sell M., Rinaldo A., Tibshirani R.J., Wasserman L. (2018). Distribution-Free Predictive Inference for Regression. J. Am. Stat. Assoc..

[4] Hulsman R. (2022). Distribution-Free Finite-Sample Guarantees and Split Conformal Prediction. Master’s Thesis.

[5] Cheon J.H., Han K., Kim A., Kim M., Song Y. (2018). Bootstrapping for Approximate Homomorphic Encryption. Advances in Cryptology—EUROCRYPT 2018.

[6] He Q., Chen J., Wu W., Feng Y. (2026). Efficient Privacy-Preserving LSTM Inference on Encrypted Sequential Data. Neurocomputing.

[7] Bakshi M., Last M. (2020). CryptoRNN: Privacy-Preserving Recurrent Neural Networks Using Homomorphic Encryption. Cyber Security Cryptography and Machine Learning.

[8] Podschwadt R., Takabi D. (2021). Non-Interactive Privacy Preserving Recurrent Neural Network Prediction with Homomorphic Encryption. 2021 IEEE 14th International Conference on Cloud Computing.

[9] Feng B., Lou Q., Jiang L., Fox G. (2021). CRYPTOGRU: Low Latency Privacy-Preserving Text Analysis With GRU. Proceedings of the 2021 Conference on Empirical Methods in Natural Language Processing.

[10] Jang J., Lee Y., Kim A., Na B., Yhee D., Lee B., Cheon J.H., Yoon S. (2022). Privacy-Preserving Deep Sequential Model with Matrix Homomorphic Encryption. Proceedings of the 2022 ACM Asia Conference on Computer and Communications Security.

[11] Wang Z., Ikeda M. (2023). High-Throughput Privacy-Preserving GRU Network with Homomorphic Encryption. 2023 International Joint Conference on Neural Networks.

[12] Wang Z., Ikeda M. (2024). Toward Bootstrapping-Free Homomorphic Encryption-Based GRU Network for Text Classification. IEEE Access.

[13] Hochreiter S., Schmidhuber J. (1997). Long Short-Term Memory. Neural Comput..

[14] Trama D., Clet P.-E., Boudguiga A., Sirdey R. (2023). Building Blocks for LSTM Homomorphic Evaluation with TFHE. Cyber Security, Cryptology, and Machine Learning.

[15] Zhang Z., Shao K., Deng R., Wang X., Zhang Y., Wang M. (2026). PrivLSTM: A Privacy-Preserving LSTM Inference Framework by Fusing Encryption and Network Structure for Multi-Sourced Data. Inf. Fusion.

[16] Paterson M.S., Stockmeyer L.J. (1973). On the Number of Nonscalar Multiplications Necessary to Evaluate Polynomials. SIAM J. Comput..

[17] Park J., Kim M.J., Jung W., Ahn J.H. (2022). AESPA: Accuracy Preserving Low-Degree Polynomial Activation for Fast Private Inference. arXiv.

[18] Ao W., Boddeti V.N. (2024). AutoFHE: Automated Adaption of CNNs for Efficient Evaluation over FHE. Proceedings of the 33rd USENIX Security Symposium, Philadelphia, PA, USA, 7–9 August 2024.

[19] Hinton G., Vinyals O., Dean J. Distilling the Knowledge in a Neural Network. Proceedings of the NIPS Deep Learning and Representation Learning Workshop.

[20] Wilks S.S. (1941). Determination of Sample Sizes for Setting Tolerance Limits. Ann. Math. Stat..

[21] Pascanu R., Mikolov T., Bengio Y. (2013). On the Difficulty of Training Recurrent Neural Networks. Proceedings of the 30th International Conference on Machine Learning.

[22] Miller J., Hardt M. (2019). Stable Recurrent Models. International Conference on Learning Representations.

[23] Lyubashevsky V., Peikert C., Regev O. (2010). On Ideal Lattices and Learning with Errors over Rings. Advances in Cryptology—EUROCRYPT 2010.

[24] Lazebnik S., Schmid C., Ponce J. (2006). Beyond Bags of Features: Spatial Pyramid Matching for Recognizing Natural Scene Categories. Proceedings of the IEEE Computer Society Conference on Computer Vision and Pattern Recognition.

[25] Halevi S., Shoup V. (2014). Algorithms in HElib. Advances in Cryptology—CRYPTO 2014.

[26] Dau H.A., Bagnall A., Kamgar K., Yeh C.C.M., Zhu Y., Gharghabi S., Ratanamahatana C.A., Keogh E. (2019). The UCR Time Series Archive. IEEE/CAA J. Autom. Sin..

[27] Tune Insight (2025). Lattigo v6.1.1: Open-Source Multiparty Homomorphic Encryption Library in Go.

[28] Bossuat J.-P., Cammarota R., Chillotti I., Curtis B.R., Dai W., Gong H., Hales E., Kim D., Kumara B., Lee C. (2025). Security Guidelines for Implementing Homomorphic Encryption. IACR Commun. Cryptol..

